# The role of microRNAs in neuroblastoma cell differentiation and their therapeutic potential: a systematic review

**DOI:** 10.3389/fonc.2026.1836015

**Published:** 2026-10-23

**Authors:** Muhammad Sulman, Kathleen E. Doyle, Amy C. Studer, Erin G. Brown

**Affiliations:** 1 School of Medicine, University of California, Davis, Davis, CA, United States; 2 Department of Surgery, University of California Davis Children’s Hospital, University of California, Davis, Davis, CA, United States; 3 Blaisdell Medical Library, University of California, Davis, Davis, CA, United States

**Keywords:** differentiation, microRNAs, mir-124, miR-17-92, miR-34, miR-506-3p, neuroblastoma, therapeutics

## Abstract

Neuroblastoma (NB) is the most common extracranial solid tumor in children under five years of age. NB tumors can arise anywhere along the sympathetic chain due to the failure of neural crest cells to appropriately migrate and differentiate. Despite aggressive multimodal therapies, high-risk NB has persistently low survival rates due to genetic heterogeneity, aggressive tumor biology, and disease relapse that is exceedingly difficult to treat. One strategy for treating NB tumors is to promote their differentiation into a more benign histologic phenotype. Retinoic acid (RA) is a vitamin A derivative that decreases cell proliferation and promotes NB cell differentiation to a more mature phenotype. RA is commonly utilized in the maintenance phase of treatment to reduce the risk of NB recurrence, but unfortunately, rates of relapse remain high. MicroRNAs (miRs) are small noncoding RNAs that regulate post-transcriptional gene expression and may promote differentiation effects in NB cells thereby representing novel therapeutics. This systematic review highlights miRs with known roles in differentiation of NB cells by discussing their major targets and pathways as well as their potential role in treatment for NB patients. Consistent with PRISMA 2020 recommendations, a systematic literature search was performed in Ovid MEDLINE and Embase databases from inception to June 2023. Articles were selected for inclusion based on relevance to the role of miRs in differentiation and NB; articles not focused on miRs, differentiation, or NB, and non-English publications were excluded. A total of 396 articles from this search underwent abstract and full-text screening to yield 89 articles matching our selection criteria. Our review revealed 53 entries comprising individual miRNAs and miRNA groups with a role in the differentiation of NB. miR-124, miR-34, and miR-506-3p are well studied with significant stimulation of NB differentiation. Disruption of these miRs is correlated with decreased overall survival in patients. The miR-17–92 cluster was found to antagonize NB differentiation and is associated with poor survival. Preclinical evidence suggests that miR-124, miR-34, and miR-506-3p families may promote differentiation of NB cells toward a more mature phenotype, though substantial translational barriers remain before clinical application.

**Systematic review registration:**

https://www.crd.york.ac.uk/PROSPERO/view/CRD42023476550, identifier CRD42023476550.

## 1 Introduction

Neuroblastoma (NB) is the most common extracranial solid tumor in children under five years old (1–3). NB tumors arise from the embryonic neural crest cells (NCCs) within the neuronal ganglia of the peripheral sympathetic nervous system. NCCs migrate away from the neural tube early in embryogenesis and can fail to differentiate appropriately, resulting in NB tumor formation in the adrenal medulla, abdominal paraspinal ganglia, and the sympathetic ganglia of the chest, head, neck, abdomen, and pelvis (4, 5). NB is classically stratified into low-, intermediate-, or high-risk disease based on the tumor’s extension or invasion into surrounding structures, the histologic and molecular features of the tumor, and other patient factors. Children with high-risk disease comprise approximately half of all cases (6, 7). The stage of the disease at the time of diagnosis is highly prognostic, with low-risk tumors in infants often showing spontaneous regression and differentiation without treatment and high-risk tumors demonstrating aggressive invasion, early metastasis, and frequent relapses despite intensive multimodal therapies (7, 8). While outcomes for patients with low or intermediate-risk disease are excellent with an overall survival greater than 90%, the overall survival for patients with high-risk disease remains dismal around 40% (5, 8).

Tumor cell differentiation plays an important role in histopathology and treatment strategy. Histopathologically, NB is characterized as: (a) undifferentiated (composed entirely of neuroblasts, no ganglion cell maturation), (b) less differentiated (predominantly neuroblasts, <5% maturing/mature ganglion cells), and (c) differentiated (predominantly neuroblasts but >5% maturing/mature ganglion cells) (9). Accelerating the differentiation process in NB tumors is one treatment strategy for patients with high-risk disease as it induces malignant cells to differentiate into mature cells, thereby leading to tumor cell growth arrest and apoptosis (10, 11). Retinoic acid (RA) remains the most efficacious agent for inducing NB differentiation (12) and 13-cis RA is currently standard of care for patients with high-risk NB. However, response to RA therapy is variable and may not be sustained after RA treatment ends (11–13). Therefore, improved understanding of the mechanism of differentiation in NB may aid in the development of new therapeutic strategies.

The histologically heterogeneous nature and wide behavioral phenotypes make NB notoriously challenging to treat. Many high-risk NB tumors have segmental chromosomal abnormalities, including deletion or loss of heterozygosity of chromosomes 1p, 11q, 14q or, alternatively, gain of chromosome 17q. Additionally, 30% of tumors have MYCN amplification, the most common oncogene associated with NB and an indicator of a worse prognosis. Numerous mutations in various oncogenic/tumor suppressive pathways involving ALK, ATRX, TP53, RAS/MAPK genes are also noted in NB (14–16). Targeting these genetic aberrations has been a long-standing goal in developing therapeutics for NB, and recently, research has characterized the role of microRNAs (miRs) in NB tumorigenesis. MicroRNAs are small (17–22 nucleotides) noncoding RNAs that regulate post-transcriptional gene expression. In general, miRs play a critical role in cellular homeostasis, and rearrangements or dysregulation in miR profiles can disrupt the balance of development, differentiation, and growth (17). MiRs play an important role in the pathogenesis of NB tumors (10) and may contribute to dysregulation of the more commonly known oncogenes associated with NB (14). Specifically, miRs regulate cellular differentiation, and understanding the precise role of miRs in NB cell differentiation may offer a novel therapeutic strategy. This systematic review aims to describe the role of miRs involved in the differentiation of NB cells and explore their therapeutic potential.

## 2 Methods

### 2.1 Search strategy and selection criteria

A systematic literature search was performed in accordance with the Preferred Reporting Items for Systematic Review and Meta-Analyses (PRISMA) statement (18). Studies were selected for inclusion based on relevance to the following PICO (Population, Intervention, Comparison, Outcome) question: in neuroblastoma, do microRNAs play a role in differentiation? (Population/problem= neuroblastoma, Intervention: microRNA, Comparison: None, Outcome: differentiation). In June 2023, we searched Ovid MEDLINE and Embase (Elsevier) databases using the following keywords and controlled vocabulary terms (MeSH, Emtree) selected to retrieve articles on microRNA, neuroblastoma, and cell differentiation: “miRNA*,” “microRNA*,” “miR-124-3p,” “miR-506-3p,” “miR-17-92”, neuroblastoma (e.g., “neuroblastoma*,” “ganglioneuroblastoma*”), and cell differentiation (e.g., “differentiation,” “reprogramming,” “maturation”). Translation of the Ovid MEDLINE search strategy to Embase was partially automated using the Polyglot Search Translator tool from SR Accelerator (19). No publication date, age, or language limits were applied in the search phase. Reproducible database search strategies are provided in the **Supplementary File 1**. Unpublished studies were not sought. Studies were selected for inclusion based on relevance to the role of miRs in differentiation and neuroblastoma, while those not focusing on miRs, differentiation, and NB were excluded. Non-English studies were also excluded during abstract screening.

MEDLINE and Embase were selected as they are the two most comprehensive biomedical databases and together provide extensive coverage of the peer-reviewed biomedical literature. We acknowledge that excluding databases such as Web of Science, Scopus, Cochrane Library, and grey literature sources (e.g., conference proceedings, preprints) may have resulted in some relevant studies being missed; however, prior methodological research has demonstrated that the combination of MEDLINE and Embase captures the vast majority of relevant studies for biomedical systematic reviews (20).

### 2.2 Data extraction and quality assessment

All abstracts were screened independently by two investigators (KD/MS) using the Covidence software. Disagreements were resolved by a third independent investigator (EB). The included abstracts underwent full-text review by the same reviewers and conflicts were resolved by the same independent third-party investigator. Each manuscript was evaluated for its relevance to the search terms and inclusion criteria.

Our query resulted in 396 potentially relevant unique articles after de-duplication. Abstract screening identified 125 articles for full-text screening after consensus review. Full-text screening of 125 articles excluded 36 that did not meet inclusion criteria—18 lacked direct evidence of microRNA-mediated differentiation, 10 were reviews or non-original studies, and 8 were non-English or retracted—yielding 89 articles for data extraction. The literature search and review process has been summarized in the PRISMA flowchart (**Figure 1**). An overview of the characteristics of the included studies is provided in **Figure 2**.

**Figure 1:**
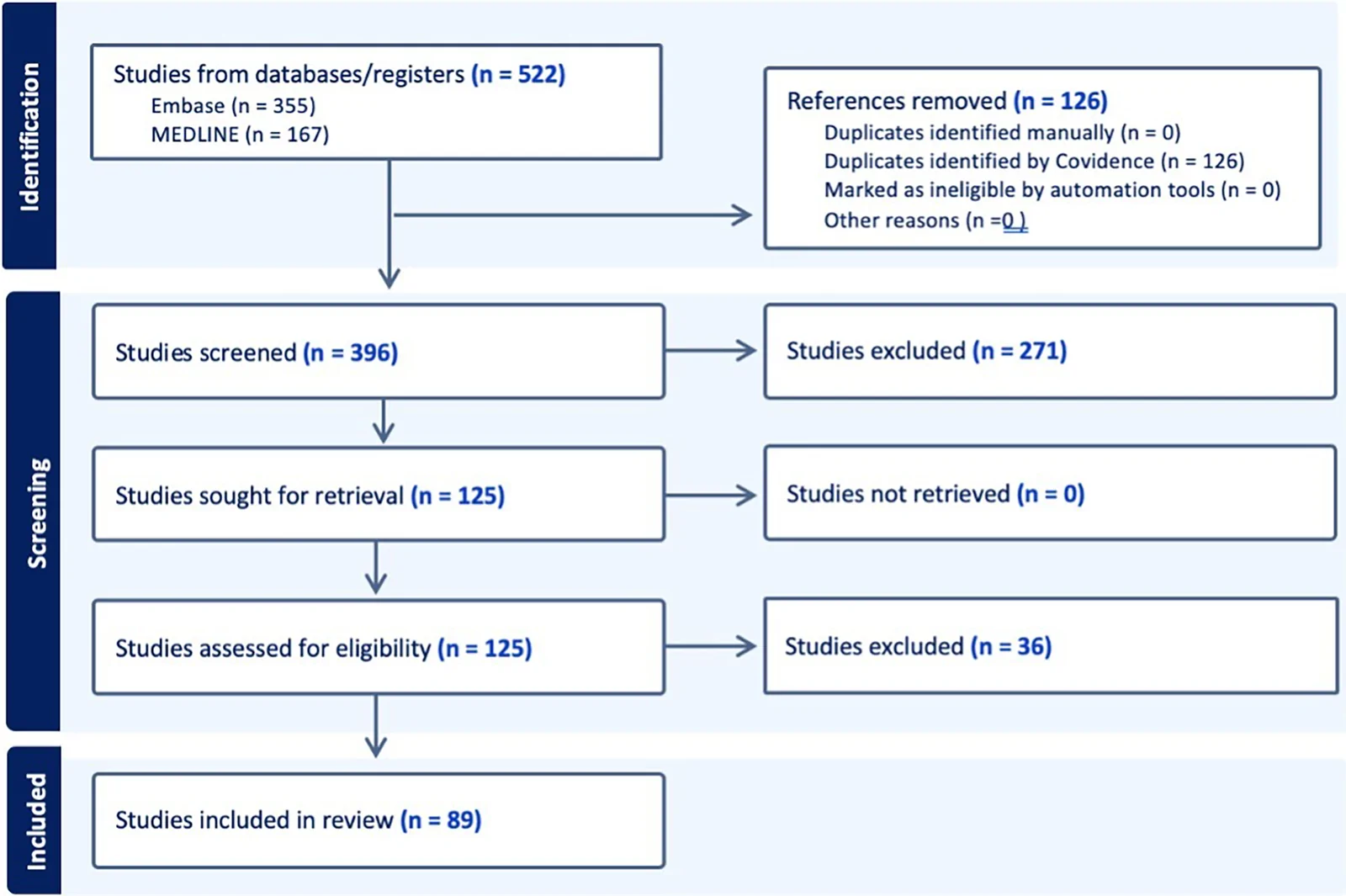
PRISMA flowchart. Following PRISMA 2020 guidelines, 396 records were initially identified from Ovid MEDLINE and Embase databases. After removing duplicates and excluding irrelevant or non-English studies, 125 full-text articles were assessed for eligibility. Of these, 89 studies met the inclusion criteria and were included in the qualitative synthesis.

**Figure 2:**
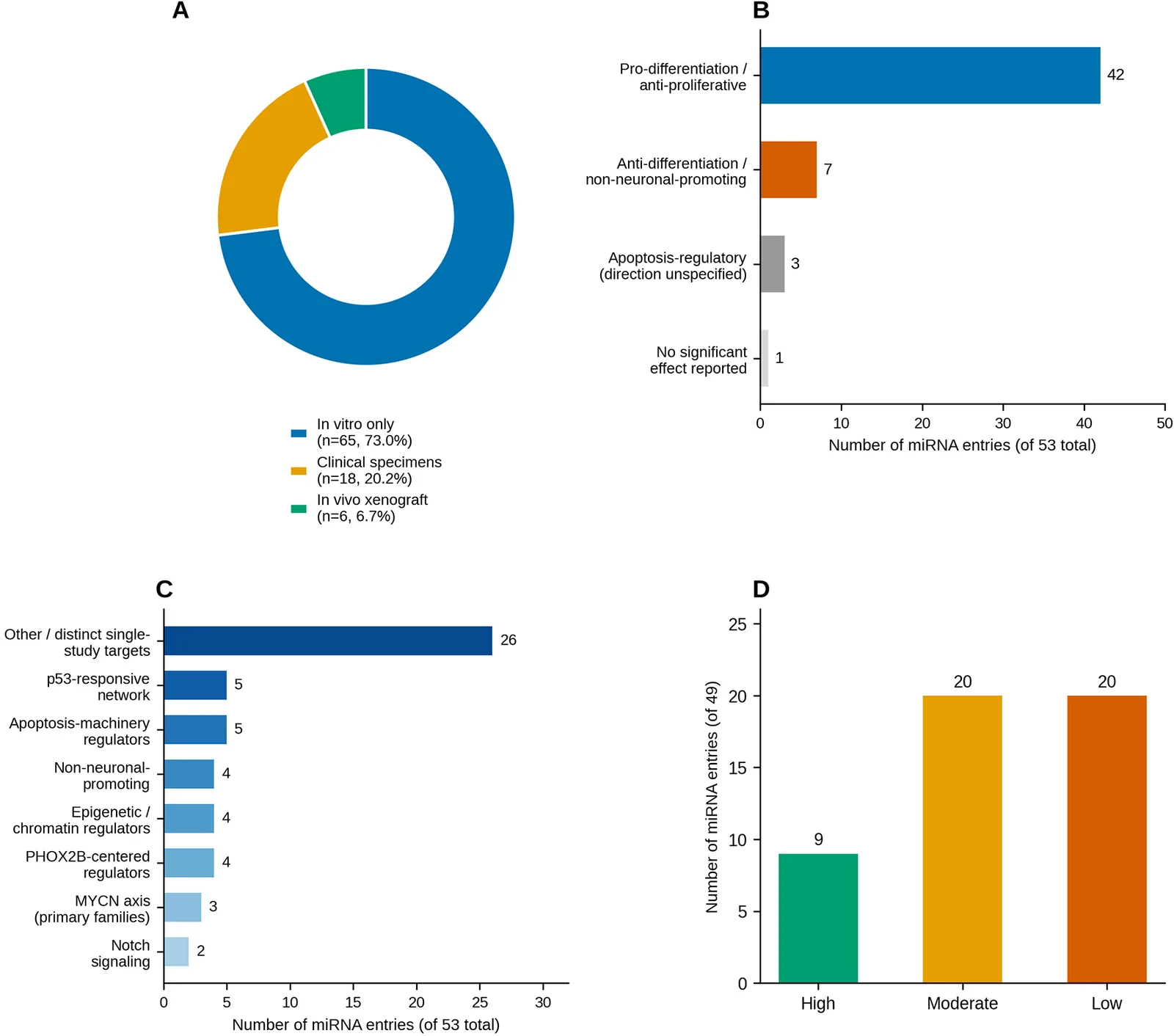
Landscape of included studies. **(A)** Distribution of experimental models across the 89 included studies. **(B)** Direction of reported functional effect across the 53 miRs/miR groups discussed (all miR discussed in this article). **(C)** Distribution of miRs by primary mechanistic/target category. **(D)** GRADE-informed quality rating distribution for the 49 entries summarized in **Table 2**.

Data extracted from each study included whether the study was conducted *in vitro* or *in vivo*, the NB cell lines used, animal models used, genes and proteins targeted by the miR(s), the overall effect on differentiation and how the differentiation was measured.

A formal risk of bias assessment using a validated instrument was not performed for the included studies. No universally validated risk of bias tool currently exists for *in vitro* studies (21), and the SYRCLE tool for animal studies (22), was applicable only to the limited subset of studies with *in vivo* components. Instead, we qualitatively assessed internal validity considerations across included studies, including the reporting of appropriate controls (e.g., scrambled miR controls, un-transfected controls), experimental replication (biological and technical replicates), cell line authentication, and consistency of findings across independent laboratories. The certainty of evidence for each outcome in the Summary of Findings table (**Table 1**) was assessed using a GRADE-informed framework adapted for preclinical evidence. In addition, each entry summarized in **Table 2** was assigned a per-miR quality rating (High/Moderate/Low) using this same GRADE-informed framework, based on replication across independent studies and breadth of experimental validation, to allow readers to directly evaluate the strength of evidence supporting each finding.

**Table 1:** MicroRNAs implicated in NB differentiation with their molecular targets and overall impact on NB cells.

MicroRNA	Molecular target	Overall impact	Experimental model	Quality (grade-informed)‡	Reference
miR-7	Downregulation of PI3K and MAPK pathways	Controls neurite outgrowth	*In vitro*	Low	(75)
Let-7	Targets RAS, MYC, HMGA2, MYCN	Suppresses tumorigenesis	*In vitro* + *In vivo*	High	(76, 77)
miR-9-2	Target differentiation inhibitor ID2, controlled by REST and CREB	Promotes differentiation and reduces proliferation	*In vitro*	Moderate	(78, 79)
miR-10a/b	Targets NCOR2 and SFRS1 (SF2/ASF	Promotes differentiation	*In vitro*	Moderate	(80, 81)
miR-21	TGFBR2	No impact on differentiation or proliferation	*In vitro*	Low	(82)
miR-23a-24-27	Target Notch1, Brn-3a/b	Regulate Differentiation	*In vitro*	Low	(83)
miR-103	Target differentiation inhibitor ID2	Promotes differentiation and reduces proliferation	*In vitro*	Low	(78)
miR-103a-3p	Inhibit N-myc	Promote differentiation	*In vitro* + clinical specimen	Moderate	(35)
miR-107	Targets BDNF, and inhibited by DLX6-AS1	Promotes differentiation and reduces proliferation	*In vitro*	Low	(84)
miR-125a	PHOX2B	reduces NB cell proliferation and augments apoptosis and differentiation	*In vitro*	Low	(85)
miR-125b	Hosted by linc-NeD125 and targets BAX and BCL-2	Limits proliferation and control apoptosis	*In vitro*	Moderate	(86, 87)
miR-127-5p	NEUROD1	suppress cell proliferation, migration and invasion	*In vitro*	Low	(88)
miR-128	Reelin, DCX, NTRK3, and BCL-2	Effects cell motility, cell growth, invasiveness, and apoptosis	*In vitro*	Moderate	(89, 90)
miR-129a	Notch1	Promotes differentiation	*In vitro*	Low	(83)
miR-130a-3p	MeCP2	Inhibits differentiation	*In vitro*	Low	(91)
miR-137	KDM1A and NEFL	Promotes differentiation	*In vitro* + clinical specimen	Moderate	(92)
miR-142	Inhibits STAU1	Inhibits differentiation	*In vitro* + *In vivo*	Moderate	(93)
miR-143-3p	Induced by p53	Upregulated in differentiation of NB and limits growth	*In vitro*	Low	(94)
miR-145	Induced by p53 and targets hypoxia-inducible factor 2 alpha	inhibits the growth, invasion, metastasis and angiogenesis	*In vitro* + *In vivo*	High	(94, 95)
miR-146a	MAP1B	Synaptic plasticity and upregulated in differentiation of NB	*In vitro*	Moderate	(94, 96)
miR-152	DNMT1, CHUK, CUL5, and GADD45A	Promote differentiation and apoptosis	*In vitro* + clinical specimen	High	(97, 98)
miR-181c	Smad7	Inhibits proliferation, migration, and invasion	*In vitro* + clinical specimen	High	(99, 100)
miR-182-5p	Induce PARP cleavage	Promotes differentiation, apoptosis, and reduces proliferation	*In vitro*	Low	(43)
miR-184	Increased Caspase-3 and caspase-7 activity	Induces cell cycle arrest and apoptosis	*In vitro* + clinical specimen	Moderate	(53)
miR-192	Induced by p53	Limits proliferation	*In vitro*	Low	(94)
miR-193	Targets DHFR	Promotes differentiation	*In vitro*	Low	(96)
miR-199	BCAT1, SMC4, RANBP3	Promotes differentiation	*In vitro*	Low	(96)
miR-200b	Inhibits ZEB1	Promotes differentiation and apoptosis	*In vitro* + clinical specimen	Moderate	(98)
miR-203a	Induced by p53	Limits proliferation	*In vitro*	Low	(43)
miR-214	Regulates endogenous Lf expression	Induces neurite growth	*In vitro*	Moderate	(75, 101)
miR-221	Correlates with MYCN amplification, HMGB1 modulates PTEN expression by transiently blocking miR-221/222	Promotes non-neuronal phenotype, downregulated during differentiation	*In vitro* + clinical specimen	High	(102, 103)
miR-222-3p	HMGB1 modulates PTEN expression by transiently blocking miR-221/222	Downregulated during differentiation	*In vitro*	Moderate	(43, 102)
miR-320d	Targets ARPP-19, HNF1A-AS1, SOX4	Induces differentiation and suppresses proliferation, migration, and invasion	*In vitro*	Low	(104)
miR-335	TGF-β, HAND1, and JAG1	Promotes non-neuronal phenotype	*In vitro* + clinical specimen	Moderate	(103)
miR-338-3p	Inhibits PTPRT and AART	Promotes differentiation and apoptosis	*In vitro* + clinical specimen	High	(98, 105, 106)
miR-340	SOX2	Upregulated during differentiation	*In vitro* + clinical specimen	Moderate	(107)
miR-375	Upregulated by N-myc to suppress Hud	Promotes neuroblast phenotype	*In vitro* + clinical specimen	Moderate	(103)
miR-376a	NFkB, FasL	Regulate NB cell death	*In vitro*	Low	(108)
miR-376c	HOX210	Induces differentiation	*In vitro* + clinical specimen	Moderate	(109)
miR-432	Induced by p53, and targets NESTIN, MeCP2 and RCOR1	Limits Proliferation, and induces differentiation	*In vitro*	Moderate	(43, 110)
miR-449a	Targets MYCN, CDK6 and LEF1.	Induces cell cycle arrest and differentiation	*In vitro* + clinical specimen	High	(35, 111)
miR-451	Targets MIF	Limits proliferation, invasion and migration	*In vitro*	Low	(112)
miR-494	Induces HO-1, HOXD9, PHOX2B	increases cell viability in NB, induces differentiation by targeting HOXD9	*In vitro* + clinical specimen	High	(109, 113)
miR-495	HOXD10, PHOX2B, BMPR1/2	Induces differentiation	*In vitro* + clinical specimen	Moderate	(109)
miR-543	PHOX2B	Induces differentiation	*In vitro* + clinical specimen	Moderate	(109)
miR-939	BcL-xl, NFkB,	Regulate NB cell death	*In vitro*	Low	(108)
miR-1290	PCNA, p27kip1 (p27), CDK5 and p35	Induce differentiation and limits cell cycle	*In vitro*	Moderate	(104, 114)
miR-2110	N-myc, TKSU	Induces differentiation and reduces cell survival	*In vitro* + clinical specimen	High	(35, 115)
miR-3613-3p	DFFB and APAF1	Downregulation of apoptosis	*In vitro*	Low	(116)

^‡^Quality was rated using a GRADE-informed framework based on (1) replication across independent studies and (2) breadth of experimental validation. “High” indicates findings replicated across ≥2 independent studies with validation extending beyond *in vitro* models (*in vivo* or clinical specimens); “Moderate” indicates either replication across ≥2 independent *in vitro* studies or a single study validated *in vivo* or in clinical specimens; “Low” indicates findings supported by a single *in vitro* study. This rating reflects the extent and consistency of available evidence rather than a formal risk-of-bias assessment of individual study methodology, consistent with the absence of a validated risk-of-bias tool for *in vitro* mechanistic studies described above.

Each entry lists the primary molecular target(s) of the miR, its overall functional impact on neuroblastoma (NB) cells—such as promotion or inhibition of differentiation, apoptosis, or proliferation—and the corresponding reference. The table highlights lesser-studied miRs and their regulatory molecules that influence NB cell fate through diverse signaling, epigenetic, and transcriptional mechanisms.

**Table 2:** Summary of findings: effects of microRNA modulation on neuroblastoma differentiation.

Population: neuroblastoma (NB) cell lines and preclinical animal models						
Outcome	No. of studies	Anticipated effect with control (no miR modulation)	Anticipated effect with miR modulation (miR-124, miR-34a, miR-506-3p overexpression or miR-17–92 inhibition)	Narrative effect estimate (range across studies)†	Direction of effect	Certainty of evidence (grade-informed)‡
Differentiation Marker Expression (βIII-tubulin, MAP2, GAP43, NSE)	12	Low to absent expression of neuronal differentiation markers	Increased expression of neuronal differentiation markers	RR range: 2.0–4.5 (fold-increase over control)	↑ Increased	⊕⊕○○ LOW — preclinical data only; consistent direction across studies
Cell Proliferation (Ki67, PCNA, cell counts)	14	Continued proliferation	Reduced proliferation, cell cycle arrest (G1/S)	RR range: 0.3–0.6 (relative reduction vs. control)	↓ Decreased	⊕⊕○○ LOW — preclinical data only; consistent direction across studies
Apoptosis Rate (Caspase-3/7, Cleaved PARP)	9	Low baseline apoptosis	Increased apoptosis markers	RR range: 1.5–4.0 (fold-increase over control)	↑ Increased	⊕⊕○○ LOW — preclinical data only; moderate consistency
Neurite Outgrowth Length	10	Minimal to no neurite extension	Significant neurite extension and network formation	MD range: 20–55 μm (increase over control)	↑ Increased	⊕⊕○○ LOW — preclinical data only; consistent direction
MYCN Expression Levels	8	High MYCN expression maintained	Reduced MYCN mRNA and protein levels	RR range: 0.2–0.5 (relative reduction vs. control)	↓ Decreased	⊕⊕○○ LOW — preclinical data only; consistent across multiple miR families

RR, relative risk (expressed as fold-change); MD, mean difference; PCNA, proliferating cell nuclear antigen; NSE, neuron-specific enolase; GAP43, growth-associated protein 43; MAP2, microtubule-associated protein 2.

^†^Values represent approximate ranges of effect sizes reported across individual studies and are NOT pooled estimates derived from meta-analysis. No formal meta-analysis was conducted due to substantial heterogeneity in study designs, cell lines, miR concentrations, transfection methods, and outcome measures across included studies. These ranges are presented to illustrate the general direction and approximate magnitude of effects and should be interpreted with caution.

^‡^Certainty of evidence was assessed using a GRADE-informed framework adapted for preclinical evidence. All outcomes start at “Low” because the evidence base is derived entirely from preclinical studies (*in vitro* and limited *in vivo*), which inherently represent a lower evidence level than clinical studies. Ratings were further adjusted based on: consistency of findings across independent laboratories, directness of outcome measures, and risk of bias considerations. “Very Low” ratings reflect outcomes with fewer contributing studies, greater variability in methods, or less direct outcome measures.

## 3 Results

Among the 89 studies included in the review, a total of 53 miRs with a role in NB differentiation were described. These 53 miRs are comprised of 49 entries in **Table 2** and the four principal miRNA groups discussed separately, with each grouped entry counted once. Additionally, the Summary of Findings table highlights the key outcomes across all included studies (**Table 1**) (23), including an overview of the anticipated absolute and relative effects of miR modulation on NB differentiation, proliferation, apoptosis, and molecular signaling. This review was conducted in accordance with the PRISMA 2020 guidelines, and the completed PRISMA checklist is provided in the **Supplementary Materials** (**Supplementary File 2**).

We identified 4 predominant miRs or miR groups as significant influencers of differentiation in NB and will focus this review by describing these miRs, their role in differentiation, their molecular mechanisms, and their clinically relevant implications in detail. The decision to focus the detailed narrative on four miRNA groups (miR-124, miR-34, miR-506-3p, and miR-17-92) is based on the following objective criteria (1): these miRNAs had the largest number of independent studies (≥5 studies each) providing sufficient evidence for detailed mechanistic discussion; (2) they had validated functional data from both gain-of-function and loss-of-function experiments; (3) they had demonstrated clinical correlations with patient outcomes; and (4) they represented both pro-differentiation and anti-differentiation mechanisms, providing a balanced perspective. Although most results were derived from *in vitro* models, convergence of findings across multiple independent studies supports a moderate level of confidence in the overall direction of effect.

### 3.1 miR-124 family

#### 3.1.1 Role in differentiation

microRNA-124 is the mature miRNA species generated from the precursor miR-124, which is encoded by three genomic loci (miR-124-1, miR-124-2, and miR-124-3) and is highly expressed in neuronal tissues, where it regulates neurogenesis, neuronal differentiation, and synaptic function. It is upregulated in NB cells following RA-induced differentiation (24), and the expression of miR-124 can promote the differentiation of neurons both *in vitro* and *in vivo* (24–26). Ectopic expression of miR-124 changes NB cell morphology from spherical, undifferentiated cells to those with extended neurites forming neuronal networks characteristic of mature neurons with increased expression levels of neuronal differentiation markers including β-Tubulin III, MAP2, SYN, NF-M, and Nestin (24, 27).

#### 3.1.2 Molecular targets/mechanisms:

Several potential mechanisms of action for the miR-124 role in differentiation have been described. First, polypyrimidine tract-binding proteins (PTBP) appear to have a role in miR-124-induced differentiation. Analysis of murine neuroblastoma cells (N2a) and mouse embryonic stem cells revealed the absence of mature miR-124 expression; instead, N2a cells expressed the immature versions of miR-124 (Pri-miR-124–1 and Pri-miR-124-2). PTBP1 binding to these Pri-miR-124–1 inhibits its maturation to miR-124, thus blocking neuronal differentiation. During N2a differentiation, the production of Pri-miR-124–1 and Pri-miR-124–2 increases. Since Pri-miR-124–2 is not inhibited by PTBP1, it matures into miR-124, which can then inhibit PTBP1 with an overall effect of promoting neuronal differentiation (28, 29). Loss of PTBP1 also removes inhibition of PTBP2, a positive regulator of N2a differentiation, allowing neuron-specific alternative splicing and initiating neuronal differentiation (28).

miR-124 also demonstrates epigenetic gene regulation by altering differentiation without changing DNA sequences. miR-124 down-regulates EZH2, a histone H3 Lys-27 histone methyltransferase, to drive cells toward neuronal differentiation (30, 31). Interestingly, miR-124-2-3p, mature miRNA strand processed from the 3’ arm of the miR-124 precursor hairpin, is found to be hypermethylated in 25% of high-risk NB samples (32) suggesting that epigenetic inhibition of miR-124 could contribute to the development of high-risk NB.

Furthermore, miR-124 modulates differentiation and the cell cycle through transcription factors, most predominantly through downregulation of CDK4, CDK6, STAT3, E2F3, and BCL2 (11, 32), thereby inducing differentiation, apoptosis, and inhibiting the cell-cycle. Additionally, miR-124 inhibits retinoic acid receptor gamma (RARG), which is implicated in downregulating neuronal outgrowth (a sign of differentiation) (33). Lastly, ELF4, a transcription factor, is the leading candidate that maintains NB in an undifferentiated state and promotes proliferation; many of the genes activated by ELF4 are repressed by miR-124 to induce differentiation (34).

miR-124 is known to interact with other miRs to form a network that promotes differentiation. Specifically, miR-124-3p networks with miR-34a-5p, miR-34b-5p, miR-506-3p, miR-449a, miR-449b-5p, miR-103a-3p, and miR-2110 to inhibit MYCN expression (35). Silencing MYCN induces differentiation, apoptosis, and growth inhibition, highlighting its important role in NB (36) (37, 38). Interestingly, the synergistic effect of using multiple miRs to generate a stronger differentiation effect is well-established and may be due to these complex miR interactions. For example, a combination of miR-124-3p, miR-128, and miR-137 downregulates MYB, PPARG, TCF3, and TCF12 in the BE (2)C cell line more effectively than any single miR (39). The interactions of miR-124 with its targets are summarized in **Figure 3**.

**Figure 3:**
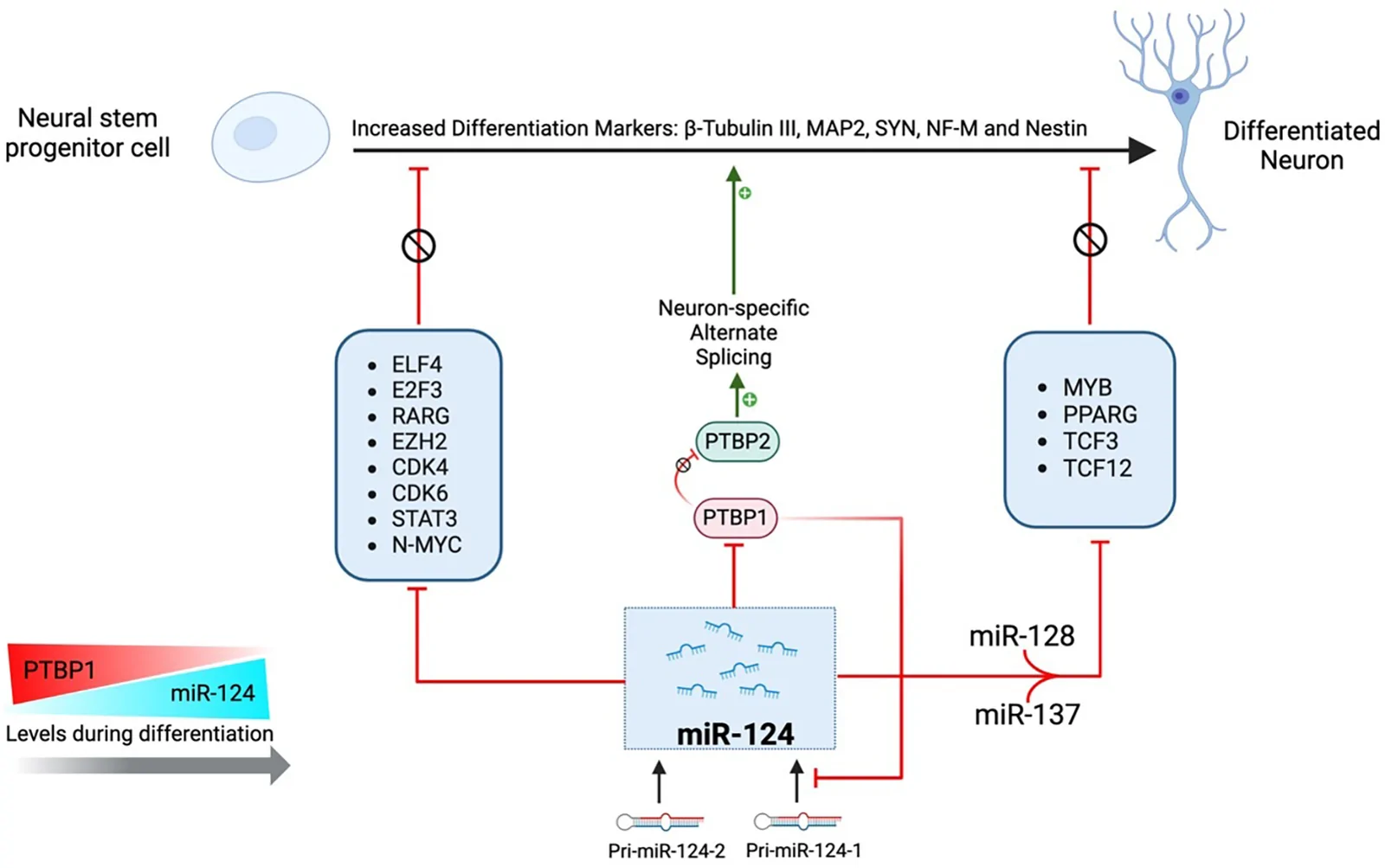
Production of miR-124 and miR-124’s interactions with its targets. Schematic summarizing how miR-124 promotes neuronal differentiation in neuroblastoma. PTBP1 binding to Pri-miR-124–1 inhibits its maturation to miR-124, thus blocking neuronal differentiation, but as differentiation progresses, Pri-miR-124–2 levels increase. pri-miR-124–2 matures into miR-124, which downregulates PTBP1, EZH2, CDK4/6, STAT3, E2F3, BCL2, RARG, and ELF4. These interactions suppress proliferation, induce apoptosis, and trigger neuronal differentiation (as noted by the increase of differentiation markers). miR-124 also acts within a microRNA network (miR-128, and miR-137) to inhibit TCF3, TCF12, PPARG, and MYB. Arrows denote activation; blunt lines indicate inhibition.

#### 3.1.3 Clinical correlation

miR-124 plays an important role in NB tumorigenesis. Low expression of the miR-124 family is linked to poor overall survival in high-risk NB patients (26, 32, 40) while high miR-124 expression has been shown to decrease NB cell viability, alter cell morphology, arrest the cell cycle, and induce apoptosis (27, 32, 41). Treatment of NB cells *in vitro* with miR-124 limited cell cycle progression, increased apoptosis, and modulated differentiation and notably, reversed treatment resistance in NB cell lines sensitized to cisplatin, a common NB chemotherapeutic (26). To date, no studies have investigated the therapeutic effects of miR-124 in human NB patients. However, current collaborative group studies are investigating the potential of miRs to serve as biomarkers for diagnosis, but also for assessing treatment response and detecting recurrence and are anticipated to have a clinical impact on NB diagnosis, risk stratification, and surveillance in the near future **Table 3**.

**Table 3:** Clinical correlations and clinical trials of microRNAs in neuroblastoma.

miR/agent	Clinical correlation in NB	Clinical trial (if applicable)	Key findings	References
miR-124 family	Low expression linked to poor OS in high-risk NB; reverses cisplatin resistance *in vitro*	None in NB; liquid biopsy studies ongoing (COG, GPOH NB2004-HR, DCOG NBL2009)	Potential biomarker for risk stratification and surveillance	(26, 32, 40, 70–72)
miR-34a	Low expression associated with high-risk NB and worse prognosis; miR-34b expression higher in >5-year survivors	MRX34 Phase I (NCT01829971) – advanced solid tumors (not NB-specific)	Trial closed early due to 4 immune-related deaths; 3 PRs and 16 SD observed; proof-of-concept for miR delivery to tumors	(32, 45, 66, 67)
miR-34a (fully modified)	N/A (preclinical)	None yet; FM-miR-34a with folate conjugation in preclinical development	Complete tumor cures in some mice; >120h target gene suppression	(63)
miR-506-3p	Absent in MYCN-amplified tumors; low expression correlates with worse prognosis	None	Truncated analogs retain activity; potential for cost-effective manufacturing	(35, 49, 50)
miR-17–92 cluster	Highly expressed in MYCN-amplified tumors; downstream target (GR) loss correlates with poor OS	None	Inhibition of cluster upregulates differentiation markers *in vitro*	(52, 55, 56)
TargomiRs (miR-16)	N/A (mesothelioma)	Phase I (NCT02369198) – malignant pleural mesothelioma	Dose-limited by inflammatory toxicity; maximum weekly dose only 5× murine therapeutic dose	(69)
miR panels (liquid biopsy)	Circulating miRs show high sensitivity for NB detection, treatment response, and relapse prediction	Incorporated into COG, GPOH, DCOG cooperative group trials	Potential to change clinical practice for diagnosis, risk stratification, and surveillance	(70–72)

OS, overall survival; PR, partial response; SD, stable disease; GR, glucocorticoid receptor; COG, Children’s oncology group; GPOH, German society for pediatric oncology and hematology; DCOG, Dutch children’s oncology group. Includes miRNAs with direct NB clinical correlations (miR-124, miR-34, miR-506-3p, miR-17-92), miRNA-based therapeutics tested in non-NB clinical trials (MRX34, TargomiRs), and emerging liquid biopsy applications. Summary of clinical associations and therapeutic trials involving miRNAs relevant to neuroblastoma (NB) differentiation.

### 3.2 miR-34 family

#### 3.2.1 Role in differentiation

The miR-34 family, consisting of miR-34a, miR-34b, and miR-34c, demonstrates tumor suppressor characteristics in various oncologic models (42). In NB, the miR-34 family plays a multifactorial role, contributing to neuronal differentiation, synaptogenesis, and cell proliferation by suppressing many cell-cycle and differentiation modulators such as the BCL2 and MYCN (32, 35).

#### 3.2.2 Molecular targets/mechanisms

Increased miR-34a levels (SH-SY5Y neuroblastoma cell line) are associated with increased levels of p53, a known tumor suppressor gene (43). TAp73, a member of the p53 family, induces miR-34a expression in differentiated NB cells. Furthermore, inhibiting either miR-34a or TAp73 reduces neurite growth during RA-induced differentiation (44),. In SK-N-BE cell line, miR-34a has also been shown to antagonize E2F3, a transcriptional inducer of cell proliferation, and decreases survivin, Cyclin D3, and C-MYC, halting cellular proliferation and promoting differentiation. NB cells treated with pre-miR-34a showed significantly increased caspase-3 and –7, which play a role in cellular apoptosis (45). Zhao et al. have also shown in human neuroblastoma specimens that miR-34a directly inhibits MYCN by targeting the MYCN 3’UTR to impair proliferation and induce differentiation of NB as well as the 3′-UTRs of polymerase I, transcript release factor and vimentin, thus highlighting the involvement of miR-34a in epithelial-mesenchymal transition and RNA transcriptional regulation (35).

Lastly, both LYAR and CTCF are targeted by miR-34a in SHEP and SH-SY5Y cells to enhance the degradation of their mRNAs to induce differentiation. DeAntonellis et al. comprehensively demonstrated the functional abilities of miR-34a to target the components of known cancer signaling cascades responsible for tumor progression and associated effects on differentiation (42). The production of miR-34a and its interactions with downstream targets are summarized in **Figure 4**.

**Figure 4:**
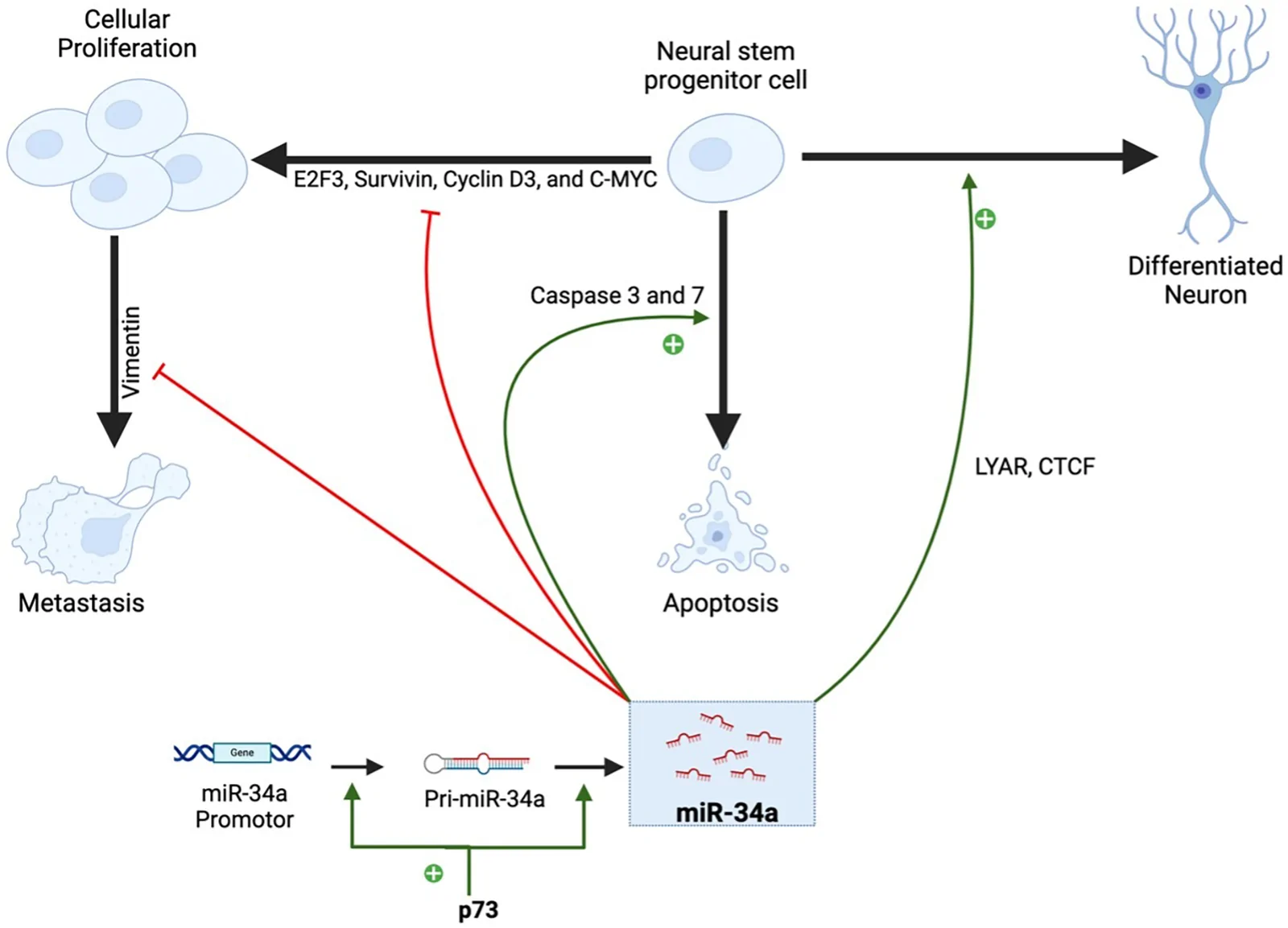
Production of miR-34a and its interactions with its downstream targets. miR-34a transcription and maturation are enhanced by p73 (member of p53 family), which in turn suppresses C-MYC, E2F3, survivin, and cyclin D3, halting proliferation and promoting neurite extension. miR-34a also enhances differentiation by targeting LYAR and CTCF for destruction. miR-34a has a crucial role in limiting metastasis by targeting vimentin.

miR-34b and miR-34c also promote the differentiation of NB cells by acting as tumor suppressors and inducing cell cycle arrest, thereby facilitating neuronal maturation. Their expression is typically suppressed in neuroblastoma due to promoter hypermethylation, and restoration of their levels—either by demethylating agents or direct transfection—leads to reduced proliferation and increased differentiation of neuroblastoma cells. Specifically, miR-34b targets cell cycle regulators such as CCNE2 and E2F3, resulting in G1 phase arrest, a prerequisite for neuronal differentiation (46, 47). Mechanistically, miR-34c is upregulated during neuronal differentiation and exerts its effects by targeting key regulators of the cell cycle and oncogenic pathways, including MYCN and BCL2, which are frequently dysregulated in neuroblastoma. Restoration of miR-34c expression in neuroblastoma cell lines leads to growth inhibition and cell cycle arrest, supporting its role in driving differentiation rather than proliferation (48).

#### 3.2.3 Clinical correlations

miR-34a is generally expressed at lower levels in high-risk NB tumors, while increased levels of miR-34b and miR-34c have been associated with improved outcomes in high-risk NB patients (45). miR-34b expression in high-risk NB tumor samples is significantly higher in patients who survived greater than 5 years compared to those whose survival was less than 5 years (32). Beyond the association of low miR-34a expression and worse prognosis in children with NB, no clinical studies have evaluated the therapeutic effect of miR-34a to date. Ongoing studies using clinical blood samples from pediatric patients with NB are investigating the role of miRs as biomarkers.

### 3.3 miR-506-3p

#### 3.3.1 Role in differentiation

miR-506-3p has become well-known for its potent differentiation-inducing effects on NB cells. Treatment with miR-506-3p results in an increase in differentiation factors βIII–tubulin, neuron-specific enolase (NSE), and growth-associated protein 43 (GAP43) (11, 35, 49, 50). miR-506-3p treatment demonstrates a dose-dependent effect on neurite outgrowth in neuroblastoma cells (BE (2)-C cell line) *in vitro* with a direct correlation between neurite outgrowth and reduction in cell viability (49).

#### 3.3.2 Molecular targets/mechanisms

miR-506-3p induces NB cell differentiation and inhibits cell cycle progression through various targets, primarily by downregulating the MYCN oncogene as noted in human NB specimens (35, 50). Increasing miR-506-3p levels decreases MYCN expression in multiple cell lines through an intermediary, pleiomorphic adenoma gene-like 2 (PLAGL2), which promotes MYCN transcription and is itself regulated by miR-506-3p. PLAGL2 knockdown induces differentiation, reduces cell proliferation, and decreases cell survival (50). PLAGL2 inhibition increases expression of neuronal differentiation markers such as βIII-tubulin, GAP43, and NSE, further supporting that high PLAGL2 levels, and by extension, high MYCN levels are associated with decreased differentiation. The same cells show decreased expression of cellular proliferation markers PCNA and Ki67 and increased expression of apoptosis marker CL PARP indicating that cellular proliferation and apoptosis are concomitantly occurring alongside differentiation (50). Combined miR-506-3p overexpression and MYCN knockdown also led to enhanced reduction of cell viability and increased differentiation markers (35), indicating that reduced cell survival is coupled with enhanced cell differentiation for the cells treated with miR506-3p even when MYCN is overexpressed.

CDK4 and STAT3 are also important proteins downregulated by miR-506-3p in BE (2)-C cells, similar to miR-124-3p (11, 32). Suppression of both STAT3 and CDK4 results in significantly greater neurite growth than suppression of either STAT3 or CDK4 alone, indicating a potential interaction between these pathways in relation to neuronal differentiation. These complex interactions between miR-506-3p and its targets are summarized in **Figure 5** below.

**Figure 5:**
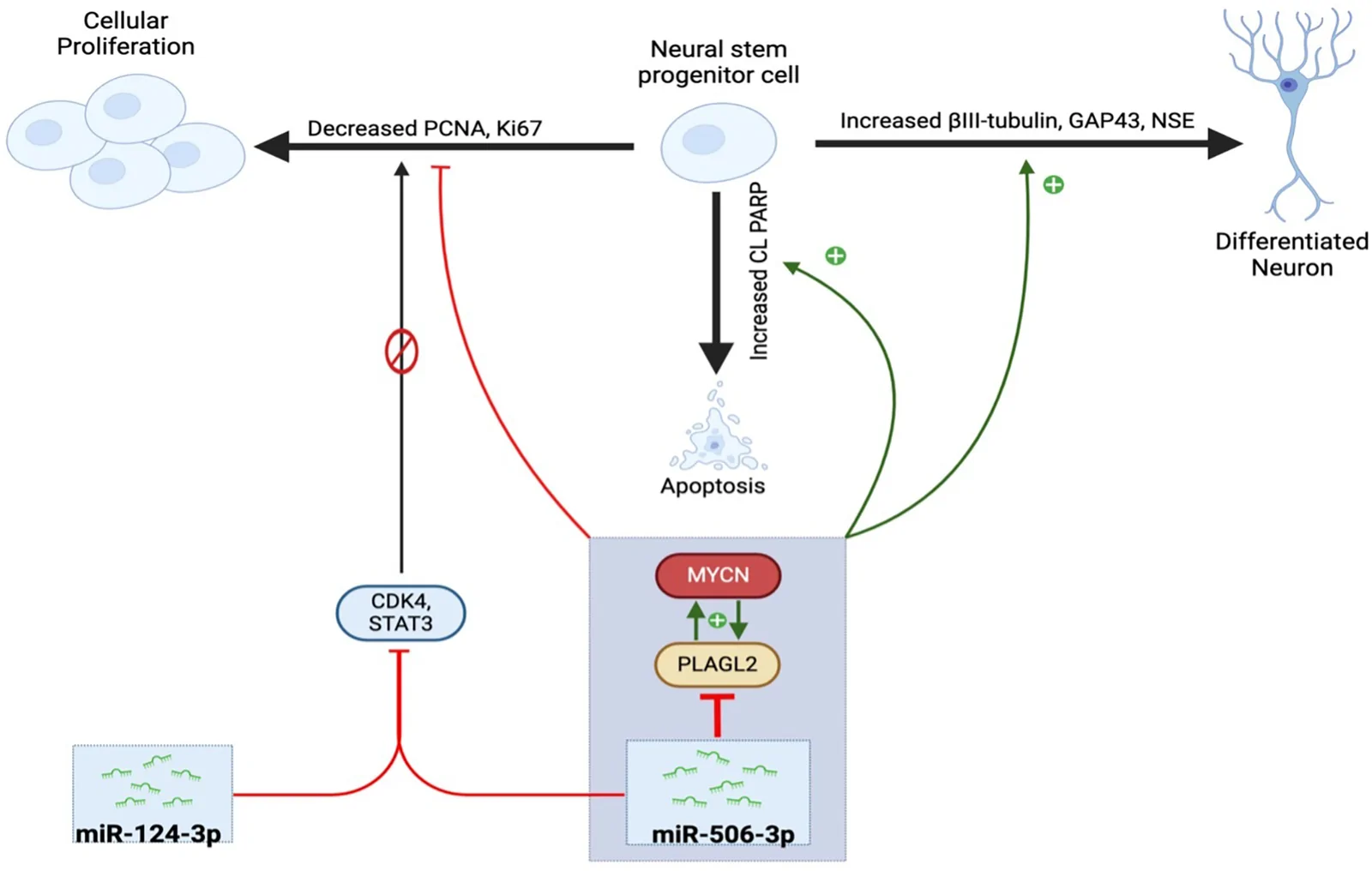
miR-506-3p and its interactions with its molecular targets. miR-506-3p induces neuroblastoma cell differentiation by targeting PLAGL2, an upstream activator of MYCN. Inhibition of PLAGL2 and MYCN increases neuronal differentiation markers (βIII-tubulin, GAP43, NSE) and decreases proliferation markers (PCNA, Ki67). Concurrent suppression of STAT3 and CDK4 in collaboration with miR-124-3p reinforces cell-cycle arrest.

#### 3.3.3 Clinical correlation

MYCN amplification is a poor prognostic indicator in NB, and there appears to be a relationship between miR-506-3p and MYCN. In MYCN-amplified tumors, miR-506-3p levels are non-existent (35). miR-506-3p regulates PLAGL2, an inducer of MYCN, and dysregulation of miR-506-3p leads to increased PLAGL2 and MYCN levels, affecting clinical outcomes (50). Thus, the expression of miR-506-3p is critical for inducing differentiation. Even truncated miR-506-3p (deleting up to two nucleotides) retains differentiation-inducing activity compared to the wild-type mimic, as shown in BE (2)-C cells (49). The shortened analog designs may lower the cost of manufacturing shortened analogs and reduce non-specific toxicity due to their smaller molecular sizes. While low miR-506-3p expression is associated with worse prognosis in children with NB, no clinical studies have evaluated the therapeutic effect of miR-506-3p for NB.

### 3.4 miR-17–92 cluster

#### 3.4.1 Role in differentiation

While many miRs induce differentiation in NB cells, some inhibit differentiation, resulting in a more aggressive phenotype with a worse prognosis. The miR-17–92 cluster (including miR-17, miR-18, miR-19, miR-20, and miR-92) is transcribed as a single polycistronic unit from one genomic locus, distinguishing it from a conventional miR family, whose members are typically encoded at separate chromosomal loci but share a common biologic function (51). This cluster is known to inhibit NB tumor differentiation, contributing to enhanced tumorigenesis and worse outcomes (52).

#### 3.4.2 Molecular targets/mechanisms

The miR-17–92 cluster, consisting of miR-17, miR-18, miR-19, miR-20, and miR-92, is downregulated upon differentiation induced by brain-derived neurotrophic factor (BDNF), which influences dendrite growth and complexity (53, 54). The miR-17–92 cluster is regulated by MYCN and directly represses genes involved in differentiation, particularly nuclear hormone receptors (NHRs) like the glucocorticoid receptor (GR) and estrogen receptor-alpha (ERα), thereby decreasing NB differentiation (**Figure 6**) (52). Reduced ERα and GR levels correlate with low TrkA, p75NTR, and SCG2 expression, the markers of differentiation (55, 56). Inhibition of the miR-17–92 cluster upregulates BCL2, MEF2D, and MAP3K12, all markers of neuronal differentiation (57). Furthermore, as shown in **Figure 6**, the miR-17–92 cluster inhibits RUNX1 and RUNX3, master transcription factors in sensory neuron lineage differentiation that are tightly controlled during tumorigenesis. The direct result of this inhibition is decreased differentiation and promotion of proliferation. Countering this inhibition has successfully induced differentiation of neuroblastoma cells *in vitro* (58).

**Figure 6:**
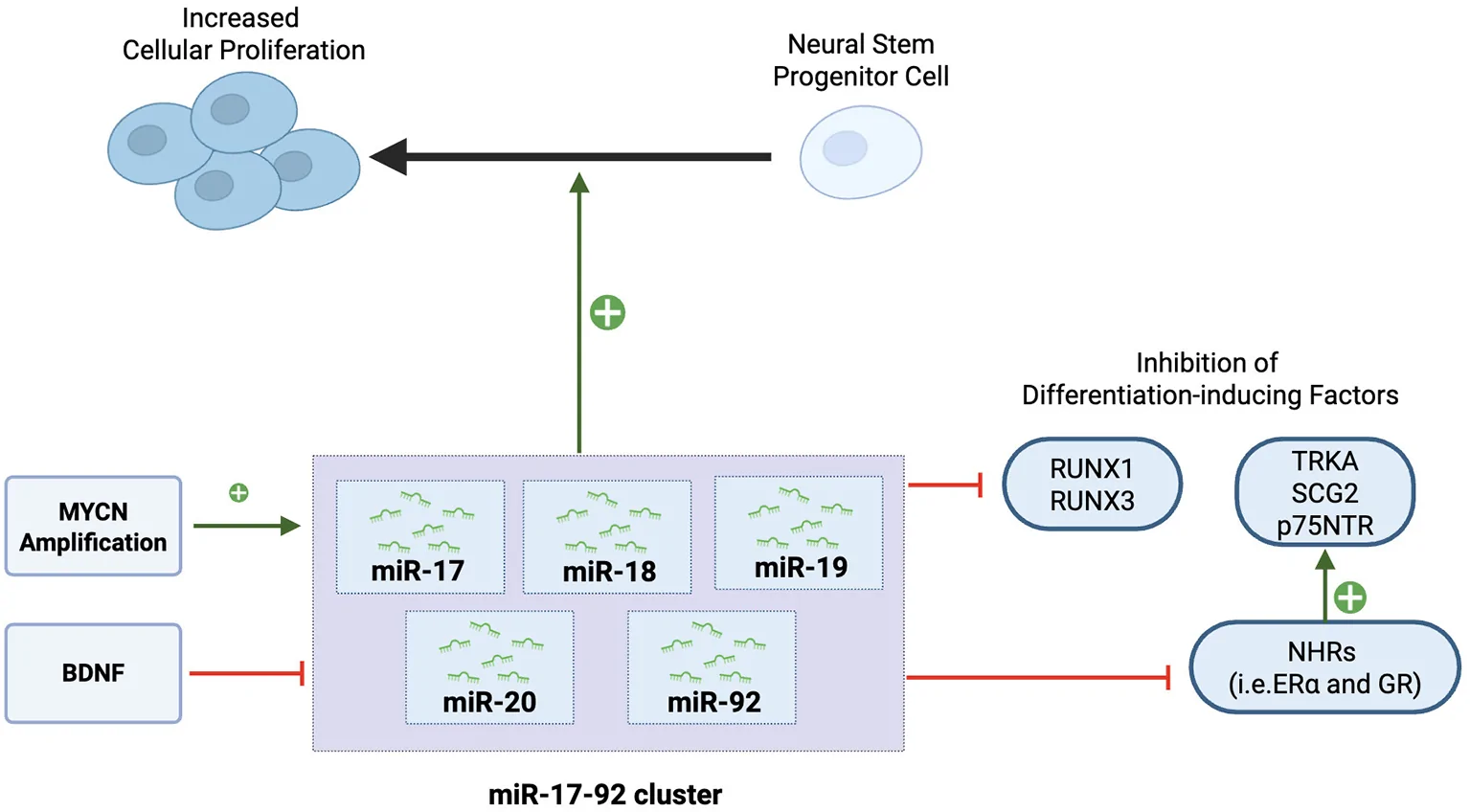
miR-17–92 cluster targets. The miR-17–92 cluster (miR-17, -18, -19, -20, -92) acts as a differentiation inhibitor under MYCN regulation. The members of miR-17–92 cluster inhibit the differentiation-inducing factors such as nuclear hormone receptors [NHRs (GR, ERα)] and transcription factors (RUNX1/3).Thus, this cluster promotes an undifferentiated, proliferative phenotype.

#### 3.4.3 Clinical correlation

Previous studies have demonstrated that the miR-17–92 cluster, especially miR-18a, is highly expressed in MYCN-amplified tumors compared to normal adrenal tissue. Furthermore, the downstream targets downregulated by the miR-17–92 cluster, such as GR expression, are positively associated with differentiation and improved overall survival in neuroblastoma patients, supporting the role of the miR-17–92 cluster in inhibiting differentiation in neuroblastoma (52, 55, 56). The prognostic role of MYCN-amplification in children with NB is clear and plays an essential role in risk stratification. The role of miR-17–92 cluster may be associated with an impact on MYCN expression and inhibition of differentiation, but no clinical studies have evaluated the therapeutic effect of miR-17–92 inhibition or its potential as a biomarker.

### 3.5 Other miRs implicated in the differentiation of NB

While the aforementioned miRs have the best characterized and understood role in NB differentiation, numerous other miRs have implications in cellular differentiation as well. These miRs are summarized in **Table 2**. Although these 49 miRs are individually less characterized than the four primary groups discussed above, they converge on a limited number of recurring regulatory themes that lend broader coherence to their collective role in NB differentiation.

A subset is directly induced by p53 activation, paralleling the p53-TAp73-miR-34a axis described above: miR-143-3p, miR-145, miR-192, miR-203a, and miR-432 are each transcriptionally upregulated downstream of p53 and largely act to limit proliferation while promoting differentiation-associated phenotypes (43, 94, 95, 110), reinforcing p53 as a broader regulatory hub for differentiation-inducing miRs beyond miR-34 alone. A second cluster converges on PHOX2B, a master transcription factor of sympathetic neuronal differentiation: miR-125a, miR-494, miR-495, and miR-543 each target or are co-regulated with PHOX2B and consistently promote differentiation-associated phenotypes (85, 109, 113), suggesting PHOX2B functions as a shared downstream node for these pro-differentiation miRs, analogous to the role MYCN plays for miR-124, miR-34, miR-506-3p, and miR-17-92. Several miRs act on chromatin and epigenetic machinery, paralleling the epigenetic mechanism described for miR-124: miR-152 targets DNMT1, miR-130a-3p and miR-432 target MeCP2, and miR-137 targets the histone demethylase KDM1A (91, 92, 97, 98, 110), each modulating differentiation through altered chromatin accessibility rather than direct transcriptional repression. A smaller group signals through Notch, including miR-23a-24–27 and miR-129a, both of which target Notch1 to promote differentiation (83), indicating that Notch pathway inhibition is a recurring, if less-studied, route to NB differentiation alongside the MYCN- and p53-centered pathways described above.

Several miRs converge on regulators of programmed cell death, including miR-125b (BAX/BCL-2) (86, 87), miR-184 (caspase-3/7) (53), miR-376a and miR-939 (NFkB/Bcl-xL/FasL) (108), and miR-3613-3p (DFFB/APAF1) (116), highlighting that differentiation and apoptosis are frequently co-regulated outcomes rather than independent processes across this literature. Notably, not all miRs in **Table 2** promote differentiation. miR-221, miR-222-3p, miR-335, and miR-375 instead favor a non-neuronal or less-differentiated neuroblast phenotype, acting largely through HMGB1/PTEN signaling or the N-myc-Hud axis (102, 103). Their inclusion is essential to a balanced synthesis of the included evidence: these findings caution against characterizing the field as uniformly pro-differentiation and instead support a bidirectional model in which specific miRs can either promote or restrain the differentiated state depending on their downstream target network. The remaining miRs in **Table 2** are each supported by a single primary study and act through distinct, largely non-overlapping targets, including BDNF (miR-107) (84), ZEB1 (miR-200b) (98), SOX2 (miR-340) (107), SOX4 (miR-320d) (104), and cell-cycle regulators such as PCNA, p27kip1, CDK5, and p35 (miR-1290) (104, 114). This diversity of targets illustrates that differentiation in NB is governed by a broad and only partially overlapping network of miR-target interactions rather than a small number of dominant pathways. Collectively, this pattern suggests that future miR-based differentiation therapies for NB may benefit from combinatorial approaches spanning several convergent pathways, including p53, PHOX2B, epigenetic regulators, and Notch signaling, rather than targeting a single miR or family in isolation. We summarize some of the major miR-regulated regulatory pathways in **Table 4**. As detailed in **Table 4**, these pathways reflect two parallel regulatory strategies that converge on the same outcome: MYCN and Notch1 signaling normally restrain differentiation and are relieved of this restraint by their converging miRs, whereas the p53/TAp73 network and PHOX2B normally promote differentiation directly and are reinforced by their associated miRs.

**Table 4:** Convergence of principal regulatory pathways on NB differentiation.

Regulatory pathway	Converging miRs	Regulatory relationship	Regulatory logic	Effect of pathway activity on differentiation‡	References
MYCN	miR-124, miR-34a, miR-506-3p, miR-449a, miR-449b, miR-103a-3p, miR-2110	Repressed by these miRs; MYCN reciprocally represses pro-differentiation miRs and drives expression of the miR-17–92 cluster	Repression-relief	Inhibits differentiation	(32, 35, 111, 115)
p53/TAp73	miR-34a, miR-143-3p, miR-145, miR-192, miR-203a, miR-432	Transcriptionally induced by p53/TAp73; represses MYCN via miR-34a	Direct induction	Promotes differentiation	(43, 94, 95, 110)
PHOX2B	miR-125a, miR-494, miR-495, miR-543	Targeted by or co-regulated with these miRs	Direct induction	Promotes differentiation	(85, 109, 113)
Notch1 signaling	miR-23a-24-27, miR-129a	Repressed by these miRs	Repression-relief	Inhibits differentiation	(83)

^‡^“Effect of Pathway Activity on Differentiation” uses a single consistent reference point throughout: the consequence of each pathway’s own intrinsic activity on NB differentiation, independent of miR-mediated regulation. These four pathways represent two parallel regulatory strategies that converge on the same outcome. MYCN and Notch1 signaling normally restrain differentiation; the miRs listed here act by repressing these pathways, thereby relieving that restraint (“repression-relief”). In contrast, the p53/TAp73 network and PHOX2B normally promote differentiation directly; the associated miRs act downstream of or alongside these pathways to reinforce that signal (“direct induction”). The p53/TAp73 and MYCN modules additionally intersect via miR-34a, which is induced by p53/TAp73 and directly represses MYCN; beyond this link, cross-talk between the four pathways has not been established in the literature reviewed here, and they are best considered largely parallel, partially overlapping routes to the same differentiation outcome.

Summary of the four principal regulatory pathways discussed throughout this review, the miRs shown to converge on each pathway, their regulatory relationship to that pathway, the underlying regulatory logic, and each pathway’s own effect on NB cell differentiation.

## 4 Discussion

NB is a heterogeneous tumor characterized by poor outcomes in patients with high-risk disease. Several important oncogenes such as MYCN, ALK, ATRX, and segmental chromosome aberrations have an important prognostic role in high-risk NB. Recent advances in immunotherapy with ALK-inhibitors such as crizotinib and lorlatinib have shown efficacy for high-risk NB and highlight the importance of ongoing investigation for new targeted therapeutic options for NB. Unfortunately, efforts to develop targeted therapeutics for MYCN have been less successful due to the complex pathways associated with gene regulation, but ongoing research to enhance understanding of the tumor microenvironment may aid therapeutic discovery.

The role of miRs in NB is extensive with key roles in tumorigenesis, metastatic potential, and differentiation (59, 60). The miRs described in this review highlight the complex roles of specific miRs and their impact on both NB cell differentiation, but also important implications for cellular apoptosis and reduced cell viability, highlighting their potential as a therapeutic adjunct for NB tumors. This comprehensive systematic review identified four predominant miR sets with critical roles in NB differentiation. Three of these miR groups, miR-124, miR-34, miR-506-3p, exhibit pro-differentiation effects and are characteristically under-expressed in high-risk NB tumors. The fourth miR group, miR-17–92 cluster demonstrates significant inhibition of NB cell differentiation and is upregulated in high-risk tumors. The miR-124 family is the most well-studied inducer of differentiation in NB; these miRs promote differentiation of neurons early in development by regulating PTBP1 expression and other transcription factors by forming a complex miR network that functions synergistically to enhance differentiation. miR-34 is well-known for its tumor-suppressing effects due to its association with Tp73 as well as its direct inhibition of MYCN, the most notorious oncogene associated with neuroblastoma. miR-506-3p also inhibits the function of MYCN through PLAGL2 to induce differentiation in neuroblastoma, while the miR-17–92 cluster promotes MYCN-regulated tumorigenesis by inhibiting nuclear hormone receptors such as GR and ER. Importantly, the miRs described in this review do not function in isolation, but rather form an interconnected regulatory network centered on key oncogenic and differentiation-related targets. MYCN serves as a central node in this network, and miR-124-3p, miR-34a-5p, miR-34b-5p, miR-506-3p, miR-449a, miR-449b-5p, miR-103a-3p, and miR-2110 all converge to inhibit MYCN expression (32). Silencing MYCN induces differentiation, apoptosis, and growth inhibition (33–35). Conversely, MYCN itself suppresses the expression of multiple differentiation-inducing miRs, creating a reciprocal feedback loop that maintains NB cells in an undifferentiated, proliferative state (53, 61). Beyond MYCN, shared downstream targets further link these miR pathways, and the synergistic effects observed when combining multiple miRs further underscore the functional interdependence of these pathways (36). This interconnected framework suggests that combinatorial miR strategies may be more effective than single-agent approaches to overcome the redundant compensatory mechanisms that characterize high-risk NB. Clinically, the role of these miRs in NB differentiation is supported by their association with overall survival. Tumors with upregulation of differentiation-inducing miRs have an improved prognosis while aggressive tumors have low levels of differentiation-inducing miRs and high levels of the miR-17–92 cluster, a potent inhibitor of differentiation. Based on the evidence reviewed, the four major miR groups function as active regulators of differentiation, not merely passive markers. This is supported by gain-of-function and loss-of-function experiments: ectopic overexpression of miR-124, miR-34a, and miR-506-3p directly induces morphological differentiation (neurite outgrowth), upregulates differentiation markers (βIII-tubulin, MAP2, GAP43, NSE), and downregulates proliferation markers (21, 24, 32, 46, 47). Conversely, inhibition of these miRs blocks RA-induced differentiation (41). Similarly, inhibition of the miR-17–92 cluster induces differentiation markers (54, 55). These functional data distinguish these miRs from biomarkers that merely correlate with differentiation status and support the potential therapeutic role for miRs as a complement to traditional NB treatment (17). The reproducibility of pro-differentiation effects for miR-124, miR-34, and miR-506-3p and the anti-differentiation effects of the miR-17–92 cluster have been independently replicated across multiple studies using different NB cell lines (e.g., SH-SY5Y, BE (2)-C, SK-N-BE, SHEP, LA-N-1), different experimental approaches (transfection of mimics, ectopic overexpression, knockdown), and by independent research groups. This convergence of findings across multiple independent studies supports a moderate level of confidence in the overall direction of effect. However, quantitative effect sizes vary across studies due to differences in cell lines, transfection efficiency, miR concentrations, and differentiation readouts. Particularly interesting is the possibility that miRs may reverse chemotherapy-related treatment resistance, as this is a key contributor to poor outcomes for high-risk NB patients and patients with relapsed disease (23). However, the studies included in this review are largely based on *in vitro* results with limited *in vivo* data, and no human trials of miR therapy for NB have been conducted to date.

The most well-characterized oncogene in high-risk NB is MYCN, conferring worse clinical prognosis when amplified. This review highlights that MYCN amplification fundamentally alters the miRNA landscape in NB and directly upregulates anti-differentiation miRs, while simultaneously suppressing pro-differentiation miRs including miR-124, miR-34a, and miR-506-3p (61, 62). In MYCN-amplified tumors, miR-506-3p expression is essentially absent (32), and miR-34a levels are significantly reduced (42). This creates a self-reinforcing oncogenic loop: MYCN suppresses differentiation-inducing miRs, and the loss of these miRs removes a key brake on MYCN expression. Importantly, both MYCN-dependent and MYCN-independent pathways mediate the differentiation-inducing function of miRs such as miR-506-3p and miR-449a, suggesting that miR-based therapies may retain efficacy regardless of MYCN status and highlighting clinical potential despite tumor heterogeneity. An enhanced understanding of the miR interactions with better-known tumor oncogenes may facilitate improved targeting of tumor oncogenes through an all-inclusive approach. This is supported by the enhanced efficacy of combination miR therapy compared to single miR agents. Furthermore, the ability of miRs to function synergistically with existing NB treatments and potentially reverse treatment resistance holds important implications for therapeutic approaches and may be a means to limit treatment toxicity for NB. Additionally, engineering approaches may enable more targeted delivery of miR therapeutics, including fully modified miR analogs that overcome stability limitations (63) and may alleviate some concerns regarding cytotoxicity (17, 64).

To date, no clinical studies have investigated the therapeutic effects of miR therapy on patients with NB, and the majority of research for miR therapeutics remains in preclinical testing. The vast majority of included studies were conducted *in vitro* using established NB cell lines (n=65, 73%), with a smaller subset conducted *in vivo* using xenograft models (n=6, 6.7%), and only a few studies analyzing clinical tumor specimens (n=18, 20.2%). It is critical to note that no clinical therapeutic trials of miRs in NB were identified; therefore, additional investigation prior to clinical translation of these studies is needed. Notably, clinical trials using miR therapeutics for oncologic treatment have been limited to melanoma, mesothelioma and other solid tumors, and significant adverse events related to cytotoxicity have resulted in withdrawal (NCT02862145) or termination (NCT01829971) (65–68). The MRX34 trial, which tested a liposomal miR-34a mimic in patients with advanced solid tumors, was terminated after serious immune-mediated adverse events resulted in four patient deaths despite dexamethasone premedication (66). Similarly, the TargomiR trial testing miR-16 mimic in mesothelioma demonstrated inflammatory toxicities that limited dosing (69). These experiences underscore the current limitations of miRNA therapeutics and highlight the critical need for improved delivery technologies. Several cooperative groups, including the Children’s Oncology Group and European high-risk NB trials (German GPOH NB2004-HR trial (70) and the Dutch DCOG NBL2009 trial (71)) have incorporated analysis of liquid biopsy for patients with NB with promising roles in diagnosis, treatment response, and surveillance being identified. MiR panels demonstrated improved sensitivity and prognostic value in predicting treatment response and risk for relapse as well as detection of recurrence compared to current surveillance approaches. Improved understanding of the clinical implications of miRs as biomarkers will only aid in expanding the foundation for miRs as potential therapeutic targets and support future research (72).

The current study is limited by the heterogenous nature of the included studies and by the inherent limitations of a systematic review. It is also important to note that the GRADE-informed quality ratings applied throughout this review (**Tables 1**, **2**) represent an evidence-certainty framework adapted for preclinical mechanistic literature, rather than a formally validated risk-of-bias instrument. These ratings should be interpreted as a structured indicator of the consistency and breadth of the available evidence for each miR or outcome, not as a formal methodological quality assessment of individual studies. While selection and publication biases likely impact the reported results, the employed search strategy is reported to capture the vast majority of relevant studies for biomedical systematic reviews. Despite the promising preclinical evidence supporting miRs as differentiation-inducing agents in NB, several significant barriers must be addressed before clinical translation. First, off-target effects and immunotoxicity pose substantial safety concerns as evidenced by clinical trials in other cancers (66, 69). To mitigate these side effects, delivery and stability remain the most critical challenges. Naked miRNA mimics are rapidly degraded by serum nucleases, cleared by the kidneys, and poorly taken up by target cells (73). While nanoparticle-based delivery systems—including lipid nanoparticles, polymeric carriers, and exosome-based platforms—have shown improved stability and tumor targeting in preclinical models (74), none have been validated in pediatric NB patients. Additionally, the predominantly *in vitro* nature of the existing NB-miR literature limits generalizability to clinical effects; however, the convergence of findings across multiple independent studies highlights the promise of miR therapy for NB and the need for further research. Future research must critically assess manufacturing scalability, cost, and regulatory hurdles for RNA-based therapeutics prior to clinical implementation. Despite these limitations, advances in targeted delivery (e.g., antibody-oligonucleotide conjugates, fully modified miRNA analogs) and the potential for combinatorial miR strategies offer a path forward for future translational research (63).

## 5 Conclusion

Despite the advancements in treating NB, outcomes for patients with high-risk NB remain dismal. Continuous genetic and molecular rearrangements contribute to the disease evolution, and treatments aimed at tumor oncogenes such as MYCN have largely been unsuccessful due to the activation of alternative and compensatory pathways. MicroRNAs have been shown to alter molecular and genetic expressions with direct implications in the differentiation of NB cells and MYCN expression. Our systematic review has identified three major miR groups (miR-124, miR-34, and miR-506-3p) with significant roles in NB cell differentiation and the miR-17–92 cluster, an inhibitor of differentiation. While the presented evidence from preclinical studies supports the potential of miRs as future therapeutics for NB treatment, substantial translational barriers remain, including delivery challenges, immunotoxicity concerns, and the lack of clinical trial data in NB patients. The successful incorporation of miR panels into cooperative group liquid biopsy studies suggests a more immediate clinical impact as biomarkers for diagnosis, risk stratification, and surveillance. Further research is warranted to develop safe and effective miR-based therapeutic strategies for this challenging disease.

## Data Availability

The original contributions presented in the study are included in the article/**Supplementary Material**, further inquiries can be directed to the corresponding author.

## References

[1] Maris JM Hogarty MD Bagatell R Cohn SL . Neuroblastoma. Lancet. (2007) 369:2106–20. doi: 10.1016/S0140-6736(07)60983-0 17586306

[2] Newman EA Abdessalam S Aldrink JH Austin M Heaton TE Bruny J . Update on neuroblastoma. J Pediatr Surg. (2019) 54:383–9. doi: 10.1016/j.jpedsurg.2018.09.004 30305231

[3] Takita J . Molecular basis and clinical features of neuroblastoma. JMA J. (2021) 4:321–31. doi: 10.31662/jmaj.2021-0077 34796286 PMC8580727

[4] Louis CU Shohet JM . Neuroblastoma: Molecular pathogenesis and therapy. Annu Rev Med. (2015) 66:49–63. doi: 10.1146/annurev-med-011514-023121 25386934 PMC4418018

[5] Park JR Eggert A Caron H . Neuroblastoma: Biology, prognosis, and treatment. Hematol Oncol Clin North Am. (2010) 24:65–86. doi: 10.1016/j.hoc.2009.11.011 20113896

[6] Castel V Grau E Noguera R Martínez F . Molecular biology of neuroblastoma. Clin Transl Oncol. (2007) 9:478–83. doi: 10.1007/s12094-007-0091-7 17720650

[7] Matthay KK Maris JM Schleiermacher G Nakagawara A Mackall CL Diller L . Neuroblastoma. Nat Rev Dis Primer. (2016) 2:16078. doi: 10.1038/nrdp.2016.78 27830764

[8] Gu Y Zhong K Peng L Ding W Zeng X . TRAF4 silencing induces cell apoptosis and improves retinoic acid sensitivity in human neuroblastoma. Neurochem Res. (2023) 48:2116–28. doi: 10.1007/s11064-023-03882-3 36795185

[9] Hazard FKGM . Neuroblastoma, ganglioneuroblastoma and ganglioneuroma (2013). Available online at: https://surgpathcriteria.stanford.edu/srbc/neuroblastoma-ganglioneuroblastoma-ganglioneuroma/printable.html (Accessed July 02, 2023).

[10] Ergin K Aktaş S Altun Z Dınız G Olgun N . MicroRNA profiles in neuroblastoma: Differences in risk and histology groups. Asia Pac J Clin Oncol. (2018) 14:e374–9. doi: 10.1111/ajco.12821 29115037

[11] Zhao Z Ma X Hsiao TH Lin G Kosti A Yu X . A high-content morphological screen identifies novel microRNAs that regulate neuroblastoma cell differentiation. Oncotarget. (2014) 5:2499–512. doi: 10.18632/oncotarget.1703 24811707 PMC4058022

[12] Zeineldin M Patel AG Dyer MA . Neuroblastoma: When differentiation goes awry. Neuron. (2022) 110:2916–28. doi: 10.1016/j.neuron.2022.07.012 35985323 PMC9509448

[13] Matthay KK Reynolds CP Seeger RC Shimada H Adkins ES Haas-Kogan D . Long-term results for children with high-risk neuroblastoma treated on a randomized trial of myeloablative therapy followed by 13- *cis* -retinoic acid: A children’s oncology group study. J Clin Oncol. (2009) 27:1007–13. doi: 10.1200/JCO.2007.13.8925 19171716 PMC2738615

[14] Fetahu IS Taschner-Mandl S . Neuroblastoma and the epigenome. Cancer Metastasis Rev. (2021) 40:173–89. doi: 10.1007/s10555-020-09946-y 33404859 PMC7897201

[15] Li Z Takenobu H Setyawati AN Akita N Haruta M Satoh S . EZH2 regulates neuroblastoma cell differentiation via NTRK1 promoter epigenetic modifications. Oncogene. (2018) 37:2714–27. doi: 10.1038/s41388-018-0133-3 29507419 PMC5955864

[16] Pugh TJ Morozova O Attiyeh EF Asgharzadeh S Wei JS Auclair D . The genetic landscape of high-risk neuroblastoma. Nat Genet. (2013) 45:279–84. doi: 10.1038/ng.2529 23334666 PMC3682833

[17] Aravindan N Subramanian K Somasundaram DB Herman TS Aravindan S . MicroRNAs in neuroblastoma tumorigenesis, therapy resistance, and disease evolution. Cancer Drug Resist. (2019) 2(4):1086–1105. doi: 10.20517/cdr.2019.68 31867575 PMC6924638

[18] Page MJ McKenzie JE Bossuyt PM Boutron I Hoffmann TC Mulrow CD . The PRISMA 2020 statement: An updated guideline for reporting systematic reviews. BMJ. (2021) 372:n71. doi: 10.1136/bmj.n71 33782057 PMC8005924

[19] Clark JM Sanders S Carter M Honeyman D Cleo G Auld Y . Improving the translation of search strategies using the Polyglot Search Translator: A randomized controlled trial. J Med Libr Assoc. (2020) 108:195–207. doi: 10.5195/jmla.2020.834 32256231 PMC7069833

[20] Bramer WM Rethlefsen ML Kleijnen J Franco OH . Optimal database combinations for literature searches in systematic reviews: A prospective exploratory study. Syst Rev. (2017) 6:245. doi: 10.1186/s13643-017-0644-y 29208034 PMC5718002

[21] Tran L Tam DNH Elshafay A Dang T Hirayama K Huy NT . Quality assessment tools used in systematic reviews of *in vitro* studies: A systematic review. BMC Med Res Methodol. (2021) 21:101. doi: 10.1186/s12874-021-01295-w 33964880 PMC8106836

[22] Hooijmans CR Rovers MM De Vries RB Leenaars M Ritskes-Hoitinga M Langendam MW . SYRCLE’s risk of bias tool for animal studies. BMC Med Res Methodol. (2014) 14:43. doi: 10.1186/1471-2288-14-43 24667063 PMC4230647

[23] Hilgart J Miles C Chase J . How to present an informative summary of findings table for systematic reviews of interventions: A tutorial. Cochrane Evid Synth Methods. (2024) 2:e12093. doi: 10.1002/cesm.12093 40475883 PMC11795893

[24] You Q Gong Q Han YQ Pi R Du YJ Dong SZ . Role of miR-124 in the regulation of retinoic acid-induced Neuro-2A cell differentiation. Neural Regener Res. (2020) 15:1133–9. doi: 10.4103/1673-5374.270417 31823894 PMC7034285

[25] Jang J Lee S Oh HJ Choi Y Choi JH Hwang DW . Fluorescence imaging of *in vivo* miR-124a-induced neurogenesis of neuronal progenitor cells using neuron-specific reporters. EJNMMI Res. (2016) 6:38. doi: 10.1186/s13550-016-0190-y 27115744 PMC4846606

[26] Nolan JC Salvucci M Carberry S Barat A Segura MF Fenn J . A context-dependent role for miR-124-3p on cell phenotype, viability and chemosensitivity in neuroblastoma *in vitro*. Front Cell Dev Biol. (2020) 8:559553. doi: 10.3389/fcell.2020.559553 33330445 PMC7714770

[27] Sharif S Ghahremani MH Soleimani M . Induction of morphological and functional differentiation of human neuroblastoma cells by miR-124. J Biosci. (2017) 42:555–63. doi: 10.1007/s12038-017-9714-5 29229874

[28] Makeyev EV Zhang J Carrasco MA Maniatis T . The microRNA miR-124 promotes neuronal differentiation by triggering brain-specific alternative pre-mRNA splicing. Mol Cell. (2007) 27:435–48. doi: 10.1016/j.molcel.2007.07.015 17679093 PMC3139456

[29] Yeom KH Mitchell S Linares AJ Zheng S Lin CH Wang XJ . Polypyrimidine tract-binding protein blocks miRNA-124 biogenesis to enforce its neuronal-specific expression in the mouse. Proc Natl Acad Sci USA. (2018) 115:E11061–70. doi: 10.1073/pnas.1809609115 30401736 PMC6255174

[30] Neo WH Yap K Lee SH Looi LS Khandelia P Neo SX . MicroRNA miR-124 controls the choice between neuronal and astrocyte differentiation by fine-tuning Ezh2 expression. J Biol Chem. (2014) 289:20788–801. doi: 10.1074/jbc.M113.525493 24878960 PMC4110287

[31] Shaw G Morse S Ararat M Graham FL . Preferential transformation of human neuronal cells by human adenoviruses and the origin of HEK 293 cells. FASEB J Off Publ Fed Am Soc Exp Biol. (2002) 16:869–71. doi: 10.1096/fj.01-0995fje 11967234

[32] Parodi F Carosio R Ragusa M Di Pietro C Maugeri M Barbagallo D . Epigenetic dysregulation in neuroblastoma: A tale of miRNAs and DNA methylation. Biochim Biophys Acta. (2016) 1859:1502–14. doi: 10.1016/j.bbagrm.2016.10.006 27751904

[33] Su X Gu X Zhang Z Li W Wang X . Retinoic acid receptor gamma is targeted by microRNA-124 and inhibits neurite outgrowth. Neuropharmacology. (2020) 163:107657. doi: 10.1016/j.neuropharm.2019.05.034 31170403

[34] Kosti A Du L Shivram H Qiao M Burns S Garcia JG . ELF4 is a target of miR-124 and promotes neuroblastoma proliferation and undifferentiated state. Mol Cancer Res MCR. (2020) 18:68–78. doi: 10.1158/1541-7786.MCR-19-0187 31624087 PMC6942226

[35] Zhao Z Ma X Shelton SD Sung DC Li M Hernandez D . A combined gene expression and functional study reveals the crosstalk between N-Myc and differentiation-inducing microRNAs in neuroblastoma cells. Oncotarget. (2016) 7:79372–87. doi: 10.18632/oncotarget.12676 27764804 PMC5346721

[36] Knoepfler PS Cheng PF Eisenman RN . N-myc is essential during neurogenesis for the rapid expansion of progenitor cell populations and the inhibition of neuronal differentiation. Genes Dev. (2002) 16:2699–712. doi: 10.1101/gad.1021202 12381668 PMC187459

[37] Kang JH Rychahou PG Ishola TA Qiao J Evers BM Chung DH . MYCN silencing induces differentiation and apoptosis in human neuroblastoma cells. Biochem Biophys Res Commun. (2006) 351:192–7. doi: 10.1016/j.bbrc.2006.10.020 17055458 PMC2708968

[38] Nara K Kusafuka T Yoneda A Oue T Sangkhathat S Fukuzawa M . Silencing of MYCN by RNA interference induces growth inhibition, apoptotic activity and cell differentiation in a neuroblastoma cell line with MYCN amplification. Int J Oncol. (2007) 1189–96. doi: 10.3892/ijo.30.5.1189 17390021

[39] Kosti A Barreiro R Guardia GDA Ostadrahimi S Kokovay E Pertsemlidis A . Synergism of proneurogenic miRNAs provides a more effective strategy to target glioma stem cells. Cancers. (2021) 13:289. doi: 10.3390/cancers13020289 33466745 PMC7831004

[40] Ghafouri-Fard S Shoorei H Bahroudi Z Abak A Majidpoor J Taheri M . An update on the role of miR-124 in the pathogenesis of human disorders. BioMed Pharmacother. (2021) 135:111198. doi: 10.1016/j.biopha.2020.111198 33412388

[41] Huang TC Chang HY Chen CY Wu PY Lee H Liao YF . Silencing of miR-124 induces neuroblastoma SK-N-SH cell differentiation, cell cycle arrest and apoptosis through promoting AHR. FEBS Lett. (2011) 585:3582–6. doi: 10.1016/j.febslet.2011.10.025 22024478

[42] De Antonellis P Carotenuto M Vandenbussche J De Vita G Ferrucci V Medaglia C . Early targets of miR-34a in neuroblastoma. Mol Cell Proteomics MCP. (2014) 13:2114–26. doi: 10.1074/mcp.M113.035808 24912852 PMC4125741

[43] Rihani A Van Goethem A Ongenaert M De Brouwer S Volders PJ Agarwal S . Genome wide expression profiling of p53 regulated miRNAs in neuroblastoma. Sci Rep. (2015) 5:9027. doi: 10.1038/srep09027 25762502 PMC4356961

[44] Agostini M Tucci P Killick R Candi E Sayan BS Rivetti di Val Cervo P . Neuronal differentiation by TAp73 is mediated by microRNA-34a regulation of synaptic protein targets. Proc Natl Acad Sci USA. (2011) 108:21093–100. doi: 10.1073/pnas.1112061109 22160687 PMC3248477

[45] Welch C Chen Y Stallings RL . MicroRNA-34a functions as a potential tumor suppressor by inducing apoptosis in neuroblastoma cells. Oncogene. (2007) 26:5017–22. doi: 10.1038/sj.onc.1210293 17297439

[46] Maugeri M Barbagallo D Barbagallo C Banelli B Di Mauro S Purrello F . Altered expression of miRNAs and methylation of their promoters are correlated in neuroblastoma. Oncotarget. (2016) 7:83330–41. doi: 10.18632/oncotarget.13090 27829219 PMC5347773

[47] Jauhari A Singh T Singh P Parmar D Yadav S . Regulation of miR-34 family in neuronal development. Mol Neurobiol. (2018) 55:936–45. doi: 10.1007/s12035-016-0359-4 28084588

[48] Cole KA Attiyeh EF Mosse YP Laquaglia MJ Diskin SJ Brodeur GM . A functional screen identifies miR-34a as a candidate neuroblastoma tumor suppressor gene. Mol Cancer Res. (2008) 6:735–42. doi: 10.1158/1541-7786.MCR-07-2102 18505919 PMC3760152

[49] Mesa-Diaz N Smith MT Cardus DF Du L . Development of shortened miR-506-3p mimics exhibiting strong differentiation-inducing activity in neuroblastoma cells. Molecules. (2023) 28:6295. doi: 10.3390/molecules28176295 37687123 PMC10489042

[50] Zhao Z Shelton SD Oviedo A Baker AL Bryant CP Omidvarnia S . The PLAGL2/MYCN/miR-506-3p interplay regulates neuroblastoma cell fate and associates with neuroblastoma progression. J Exp Clin Cancer Res CR. (2020) 39:41. doi: 10.1186/s13046-020-1531-2 32087738 PMC7036248

[51] Olive V Li Q He L . mir‐17‐92: a polycistronic oncomir with pleiotropic functions. Immunol Rev. (2013) 253:158–66. doi: 10.1111/imr.12054 23550645 PMC3972423

[52] Ribeiro D Klarqvist MDR Westermark UK Oliynyk G Dzieran J Kock A . Regulation of nuclear hormone receptors by MYCN-driven miRNAs impacts neural differentiation and survival in neuroblastoma patients. Cell Rep. (2016) 16:979–93. doi: 10.1016/j.celrep.2016.06.052 27396325

[53] Chen Y Stallings RL . Differential patterns of microRNA expression in neuroblastoma are correlated with prognosis, differentiation, and apoptosis. Cancer Res. (2007) 67:976–83. doi: 10.1158/0008-5472.CAN-06-3667 17283129

[54] Goldie BJ Barnett MM Cairns MJ . BDNF and the maturation of posttranscriptional regulatory networks in human SH-SY5Y neuroblast differentiation. Front Cell Neurosci. (2014) 8:325. doi: 10.3389/fncel.2014.00325 25360083 PMC4197648

[55] Dzieran J Rodriguez Garcia A Westermark UK Henley AB Eyre Sánchez E Träger C . *MYCN* -amplified neuroblastoma maintains an aggressive and undifferentiated phenotype by deregulation of estrogen and NGF signaling. Proc Natl Acad Sci. (2018) 115:1229–38. doi: 10.1073/pnas.1710901115 29374092 PMC5819392

[56] Sainero-Alcolado L Mushtaq M Liaño-Pons J Rodriguez-Garcia A Yuan Y Liu T . Expression and activation of nuclear hormone receptors result in neuronal differentiation and favorable prognosis in neuroblastoma. J Exp Clin Cancer Res CR. (2022) 41:226. doi: 10.1186/s13046-022-02399-x 35850708 PMC9295514

[57] Beveridge NJ Tooney PA Carroll AP Tran N Cairns MJ . Down-regulation of miR-17 family expression in response to retinoic acid induced neuronal differentiation. Cell Signal. (2009) 21:1837–45. doi: 10.1016/j.cellsig.2009.07.019 19666108

[58] Inoue K . N-Myc-activated microRNAs inhibit protein synthesis of RUNX1 and RUNX3 in neuroblastoma cell lines. Dokkyo J Med Sci. (2018) 45.2:43–51. Available online at: https://dmu.repo.nii.ac.jp/record/2114/files/DJMS-45-2-2.pdf (Accessed July 16, 2024).

[59] Leichter AL Sullivan MJ Eccles MR Chatterjee A . MicroRNA expression patterns and signalling pathways in the development and progression of childhood solid tumours. Mol Cancer. (2017) 16:15. doi: 10.1186/s12943-017-0584-0 28103887 PMC5248531

[60] Salomão KB Pezuk JA De Souza GR Chagas P Pereira TC Valera ET . MicroRNA dysregulation interplay with childhood abdominal tumors. Cancer Metastasis Rev. (2019) 38:783–811. doi: 10.1007/s10555-019-09829-x 31848768

[61] Bray I Bryan K Prenter S Buckley PG Foley NH Murphy DM . Widespread dysregulation of miRNAs by MYCN amplification and chromosomal imbalances in neuroblastoma: Association of miRNA expression with survival. PloS One. (2009) 4:e7850. doi: 10.1371/journal.pone.0007850 19924232 PMC2773120

[62] Misiak D Hagemann S Bell JL Busch B Lederer M Bley N . The microRNA landscape of MYCN-amplified neuroblastoma. Front Oncol. (2021) 11:647737. doi: 10.3389/fonc.2021.647737 34026620 PMC8138323

[63] Abdelaal AM Sohal IS Iyer S Sudarshan K Kothandaraman H Lanman NA . A first-in-class fully modified version of miR-34a with outstanding stability, activity, and anti-tumor efficacy. Oncogene. (2023) 42:2985–99. doi: 10.1038/s41388-023-02801-8 37666938 PMC10541324

[64] Brillante S Volpe M Indrieri A . Advances in microRNA therapeutics: From preclinical to clinical studies. Hum Gene Ther. (2024) 35:628–48. doi: 10.1089/hum.2024.113 39150011

[65] Desantis V Saltarella I Lamanuzzi A Melaccio A Solimando AG Mariggiò MA . MicroRNAs-based nano-strategies as new therapeutic approach in multiple myeloma to overcome disease progression and drug resistance. Int J Mol Sci. (2020) 21:3084. doi: 10.3390/ijms21093084 32349317 PMC7247691

[66] Hong DS Kang YK Borad M Sachdev J Ejadi S Lim HY . Phase 1 study of MRX34, a liposomal miR-34a mimic, in patients with advanced solid tumours. Br J Cancer. (2020) 122:1630–7. doi: 10.1038/s41416-020-0802-1 32238921 PMC7251107

[67] Beg MS Brenner AJ Sachdev J Borad M Kang YK Stoudemire J . Phase I study of MRX34, a liposomal miR-34a mimic, administered twice weekly in patients with advanced solid tumors. Invest New Drugs. (2017) 35:180–8. doi: 10.1007/s10637-016-0407-y 27917453 PMC5893501

[68] Seyhan AA . Trials and tribulations of microRNA therapeutics. Int J Mol Sci. (2024) 25:1469. doi: 10.3390/ijms25031469 38338746 PMC10855871

[69] Van Zandwijk N Pavlakis N Kao SC Linton A Boyer MJ Clarke S . Safety and activity of microRNA-loaded minicells in patients with recurrent malignant pleural mesothelioma: a first-in-man, phase 1, open-label, dose-escalation study. Lancet Oncol. (2017) 18:1386–96. doi: 10.1016/S1470-2045(17)30621-6 28870611

[70] Berthold F Faldum A Ernst A Boos J Dilloo D Eggert A . Extended induction chemotherapy does not improve the outcome for high-risk neuroblastoma patients: results of the randomized open-label GPOH trial NB2004-HR. Ann Oncol. (2020) 31:422–9. doi: 10.1016/j.annonc.2019.11.011 32067684

[71] Van Zogchel LMJ Decarolis B Van Wezel EM Zappeij‐Kannegieter L Gelineau NU Schumacher‐Kuckelkorn R . Sensitive liquid biopsy monitoring correlates with outcome in the prospective international GPOH-DCOG high-risk neuroblastoma RT-qPCR validation study. J Exp Clin Cancer Res. (2024) 43:331. doi: 10.1186/s13046-024-03261-y 39722049 PMC11670466

[72] Singh J Peters NJ Avti P Trehan A Mahajan JK Menon P . The role of liquid biopsy in neuroblastoma: A scoping review. J Pediatr Surg. (2025) 60:161887. doi: 10.1016/j.jpedsurg.2024.161887 39294087

[73] Wang H Jiang Y Peng H Chen Y Zhu P Huang Y . Recent progress in microRNA delivery for cancer therapy by non-viral synthetic vectors. Adv Drug Delivery Rev. (2015) 81:142–60. doi: 10.1016/j.addr.2014.10.031 25450259

[74] Kuran SA Adıyıl R Zıkşahna K Özkan M Ihlamur M . Nanotechnology-enabled miRNA delivery systems next-generation molecular strategies in cancer therapy. Biochem Biophys Res Commun. (2025) 792:152923. doi: 10.1016/j.bbrc.2025.152923 41242300

[75] Chen H Shalom-Feuerstein R Riley J Zhang SD Tucci P Agostini M . miR-7 and miR-214 are specifically expressed during neuroblastoma differentiation, cortical development and embryonic stem cells differentiation, and control neurite outgrowth *in vitro*. Biochem Biophys Res Commun. (2010) 394:921–7. doi: 10.1016/j.bbrc.2010.03.076 20230785

[76] Molenaar JJ Domingo-Fernández R Ebus ME Lindner S Koster J Drabek K . LIN28B induces neuroblastoma and enhances MYCN levels via let-7 suppression. Nat Genet. (2012) 44:1199–206. doi: 10.1038/ng.2436 23042116

[77] Powers JT Tsanov KM Pearson DS Roels F Spina CS Ebright R . Multiple mechanisms disrupt the let-7 microRNA family in neuroblastoma. Nature. (2016) 535:246–51. doi: 10.1038/nature18632 27383785 PMC4947006

[78] Annibali D Gioia U Savino M Laneve P Caffarelli E Nasi S . A new module in neural differentiation control: two microRNAs upregulated by retinoic acid, miR-9 and -103, target the differentiation inhibitor ID2. PloS One. (2012) 7:e40269. doi: 10.1371/journal.pone.0040269 22848373 PMC3405103

[79] Laneve P Gioia U Andriotto A Moretti F Bozzoni I Caffarelli E . A minicircuitry involving REST and CREB controls miR-9-2 expression during human neuronal differentiation. Nucleic Acids Res. (2010) 38:6895–905. doi: 10.1093/nar/gkq604 20624818 PMC2978373

[80] Foley NH Bray I Watters KM Das S Bryan K Bernas T . MicroRNAs 10a and 10b are potent inducers of neuroblastoma cell differentiation through targeting of nuclear receptor corepressor 2. Cell Death Differ. (2011) 18:1089–98. doi: 10.1038/cdd.2010.172 21212796 PMC3114164

[81] Meseguer S Mudduluru G Escamilla JM Allgayer H Barettino D . MicroRNAs-10a and -10b contribute to retinoic acid-induced differentiation of neuroblastoma cells and target the alternative splicing regulatory factor SFRS1 (SF2/ASF). J Biol Chem. (2011) 286:4150–64. doi: 10.1074/jbc.M110.167817 21118818 PMC3039334

[82] Buechner J Henriksen JR Haug BH Tømte E Flaegstad T Einvik C . Inhibition of mir-21, which is up-regulated during MYCN knockdown-mediated differentiation, does not prevent differentiation of neuroblastoma cells. Differ Res Biol Divers. (2011) 81:25–34. doi: 10.1016/j.diff.2010.09.184 20980091

[83] Fukuda Y Kawasaki H Taira K . Exploration of human miRNA target genes in neuronal differentiation. Nucleic Acids Symp Ser 2004. (2005) (49)341–2. doi: 10.1093/nass/49.1.341 17150773

[84] Zhang H Xing M Guo J Zhao J Chen X Jiang Z . Long noncoding RNA DLX6-AS1 promotes neuroblastoma progression by regulating miR-107/BDNF pathway. Cancer Cell Int. (2019) 19:313. doi: 10.1186/s12935-019-0968-x 31787850 PMC6880520

[85] Liu D Tang X Huang Z Wen J Zhou Y . Histone deacetylase HDAC2 regulates microRNA-125a expression in neuroblastoma. Brain Behav. (2022) 12:e2401. doi: 10.1002/brb3.2401 35060363 PMC8865159

[86] Bevilacqua V Gioia U Di Carlo V Tortorelli AF Colombo T Bozzoni I . Identification of linc-NeD125, a novel long non coding RNA that hosts miR-125b-1 and negatively controls proliferation of human neuroblastoma cells. RNA Biol. (2015) 12:1323–37. doi: 10.1080/15476286.2015.1096488 26480000 PMC4829289

[87] Le MTN Xie H Zhou B Chia PH Rizk P Um M . MicroRNA-125b promotes neuronal differentiation in human cells by repressing multiple targets. Mol Cell Biol. (2009) 29:5290–305. doi: 10.1128/MCB.01694-08 19635812 PMC2747988

[88] Yang J Liu B Xu Z Feng M . Silencing of Circ_0135889 restrains proliferation and tumorigenicity of human neuroblastoma cells. J Surg Res. (2022) 279:135–47. doi: 10.1016/j.jss.2022.05.025 35772185

[89] Evangelisti C Florian MC Massimi I Dominici C Giannini G Galardi S . MiR-128 up-regulation inhibits Reelin and DCX expression and reduces neuroblastoma cell motility and invasiveness. FASEB J Off Publ Fed Am Soc Exp Biol. (2009) 23:4276–87. doi: 10.1096/fj.09-134965 19713529

[90] Guidi M Muiños-Gimeno M Kagerbauer B Martí E Estivill X Espinosa-Parrilla Y . Overexpression of miR-128 specifically inhibits the truncated isoform of NTRK3 and upregulates BCL2 in SH-SY5Y neuroblastoma cells. BMC Mol Biol. (2010) 11:95. doi: 10.1186/1471-2199-11-95 21143953 PMC3019150

[91] Wei B Xiao G Wu C Xu Y . HAGLR promotes neuron differentiation through the miR-130a-3p-MeCP2 axis. Open Med. (2021) 16:1121–31. doi: 10.1515/med-2021-0301 34430707 PMC8345017

[92] Althoff K Beckers A Odersky A Mestdagh P Köster J Bray IM . MiR-137 functions as a tumor suppressor in neuroblastoma by downregulating KDM1A. Int J Cancer. (2013) 133:1064–73. doi: 10.1002/ijc.28091 23400681

[93] Oh Y Park J Kim JI Chang MY Lee SH Cho YH . Lin28B and miR-142-3p regulate neuronal differentiation by modulating Staufen1 expression. Cell Death Differ. (2018) 25:432–43. doi: 10.1038/cdd.2017.182 29099484 PMC5762855

[94] Jauhari A Singh T Pandey A Singh P Singh N Srivastava AK . Differentiation induces dramatic changes in miRNA profile, where loss of Dicer diverts differentiating SH-SY5Y cells toward senescence. Mol Neurobiol. (2017) 54:4986–95. doi: 10.1007/s12035-016-0042-9 27525675

[95] Zhang H Pu J Qi T Qi M Yang C Li S . MicroRNA-145 inhibits the growth, invasion, metastasis and angiogenesis of neuroblastoma cells through targeting hypoxia-inducible factor 2 alpha. Oncogene. (2014) 33:387–97. doi: 10.1038/onc.2012.574 23222716

[96] Pandey A Sarkar S Yadav SK Yadav SS Srikrishna S Siddiqui MH . Studies on regulation of global protein profile and cellular bioenergetics of differentiating SH-SY5Y cells. Mol Neurobiol. (2022) 59:1799–818. doi: 10.1007/s12035-021-02667-5 35025051

[97] Das S Foley N Bryan K Watters KM Bray I Murphy DM . MicroRNA mediates DNA demethylation events triggered by retinoic acid during neuroblastoma cell differentiation. Cancer Res. (2010) 70:7874–84. doi: 10.1158/0008-5472.CAN-10-1534 20841484 PMC2955783

[98] Ragusa M Majorana A Banelli B Barbagallo D Statello L Casciano I . MIR152, MIR200B, and MIR338, human positional and functional neuroblastoma candidates, are involved in neuroblast differentiation and apoptosis. J Mol Med Berl Ger. (2010) 88:1041–53. doi: 10.1007/s00109-010-0643-0 20574809

[99] Li Y Wang H Li J Yue W . MiR-181c modulates the proliferation, migration, and invasion of neuroblastoma cells by targeting Smad7. Acta Biochim Biophys Sin. (2014) 46:48–55. doi: 10.1093/abbs/gmt124 24345480

[100] Xu X Wu J Ren G Hu Q . miR-181c expression in neuroblastoma children and proliferation of neuroblastoma M17 cells. Oncol Lett. (2019) 18(3):3025–30. doi: 10.3892/ol.2019.10602 31402960 PMC6676707

[101] Sriramoju B Kanwar RK Kanwar JR . Lactoferrin induced neuronal differentiation: A boon for brain tumours. Int J Dev Neurosci Off J Int Soc Dev Neurosci. (2015) 41:28–36. doi: 10.1016/j.ijdevneu.2014.12.005 25498991

[102] Mari E Zicari A Fico F Massimi I Martina L Mardente S . Action of HMGB1 on miR-221/222 cluster in neuroblastoma cell lines. Oncol Lett. (2016) 12:2133–8. doi: 10.3892/ol.2016.4876 27602152 PMC4998517

[103] Samaraweera L Grandinetti KB Huang R Spengler BA Ross RA . MicroRNAs define distinct human neuroblastoma cell phenotypes and regulate their differentiation and tumorigenicity. BMC Cancer. (2014) 14:309. doi: 10.1186/1471-2407-14-309 24885481 PMC4038381

[104] Kim YS Gong X Rubin LP Choi SW Kim Y . β-Carotene 15,15’-oxygenase inhibits cancer cell stemness and metastasis by regulating differentiation-related miRNAs in human neuroblastoma. J Nutr Biochem. (2019) 69:31–43. doi: 10.1016/j.jnutbio.2019.03.010 31048207

[105] Barik S . An intronic microRNA silences genes that are functionally antagonistic to its host gene. Nucleic Acids Res. (2008) 36:5232–41. doi: 10.1093/nar/gkn513 18684991 PMC2532712

[106] Kos A Olde Loohuis NFM Wieczorek ML Glennon JC Martens GJM Kolk SM . A potential regulatory role for intronic microRNA-338-3p for its host gene encoding apoptosis-associated tyrosine kinase. PloS One. (2012) 7:e31022. doi: 10.1371/journal.pone.0031022 22363537 PMC3281898

[107] Das S Bryan K Buckley PG Piskareva O Bray IM Foley N . Modulation of neuroblastoma disease pathogenesis by an extensive network of epigenetically regulated microRNAs. Oncogene. (2013) 32:2927–36. doi: 10.1038/onc.2012.311 22797059 PMC3477279

[108] Cui C Yu J Huang S Zhu H Huang Z . Transcriptional regulation of gene expression by microRNAs as endogenous decoys of transcription factors. Cell Physiol Biochem Int J Exp Cell Physiol Biochem Pharmacol. (2014) 33:1698–714. doi: 10.1159/000362952 24923223

[109] Bagci O Tumer S Altungoz O . Chromosome 1p status in neuroblastoma correlates with higher expression levels of miRNAs targeting neuronal differentiation pathway. In Vitro Cell Dev Biol Anim. (2023) 59:100–8. doi: 10.1007/s11626-023-00750-w 36800078

[110] Das E Bhattacharyya NP . MicroRNA-432 contributes to dopamine cocktail and retinoic acid induced differentiation of human neuroblastoma cells by targeting NESTIN and RCOR1 genes. FEBS Lett. (2014) 588:1706–14. doi: 10.1016/j.febslet.2014.03.015 24657437

[111] Zhao Z Ma X Sung D Li M Kosti A Lin G . microRNA-449a functions as a tumor suppressor in neuroblastoma through inducing cell differentiation and cell cycle arrest. RNA Biol. (2015) 12:538–54. doi: 10.1080/15476286.2015.1023495 25760387 PMC4615578

[112] Liu G Xu Z Hao D . MicroRNA-451 inhibits neuroblastoma proliferation, invasion and migration by targeting macrophage migration inhibitory factor. Mol Med Rep. (2016) 13:2253–60. doi: 10.3892/mmr.2016.4770 26783235

[113] Piras S Furfaro AL Caggiano R Brondolo L Garibaldi S Ivaldo C . microRNA-494 favors HO-1 expression in neuroblastoma cells exposed to oxidative stress in a Bach1-independent way. Front Oncol. (2018) 8:199. doi: 10.3389/fonc.2018.00199 29951371 PMC6008388

[114] Yelamanchili SV Morsey B Harrison EB Rennard DA Emanuel K Thapa I . The evolutionary young miR-1290 favors mitotic exit and differentiation of human neural progenitors through altering the cell cycle proteins. Cell Death Dis. (2014) 5:e982. doi: 10.1038/cddis.2013.498 24407235 PMC4040694

[115] Zhao Z Partridge V Sousares M Shelton SD Holland CL Pertsemlidis A . microRNA-2110 functions as an onco-suppressor in neuroblastoma by directly targeting Tsukushi. PloS One. (2018) 13:e0208777. doi: 10.1371/journal.pone.0208777 30550571 PMC6294380

[116] Boratyn E Nowak I Horwacik I Durbas M Mistarz A Kukla M . Monocyte chemoattractant protein-induced protein 1 overexpression modulates transcriptome, including microRNA, in human neuroblastoma cells. J Cell Biochem. (2016) 117:694–707. doi: 10.1002/jcb.25354 26308737

