# Supplementary material for: The role of microRNAs in neuroblastoma cell differentiation and their therapeutic potential: a systematic review
Source: Front Oncol. 2026 Oct 23;16:1836015. doi: 10.3389/fonc.2026.1836015 (PMC13739540; doi:10.3389/fonc.2026.1836015)
Supplement: Supplementary file 1 [file Table1.docx]

**Search Strategy:**

Ovid MEDLINE(R) ALL <1946 to June 09, 2023>

Run date: June 12, 2023

Results: 167

1	exp MicroRNAs/

2	(miRNA* or microRNA* or micro RNA* or miRNome*).ab,kf,ti.

3	(miR-2110 or miR-340 or miR-506-3p or miR-124-3p or miR-61 or miR-10A or miR-10B or miR-14q32 or miR-15a or miR-15b or miR-

16 or miR-17-92 or miR137 or miR-410 or miR-487b or miR-490-5p or miR-558 or miR-9-3p or miR-218-5p or miR-1538).ab,kf,ti.

4	1 or 2 or 3

5	exp Neuroblastoma/

6	(Neuroblastoma* or ganglioneuroblastoma*).ab,kf,ti.

7	5 or 6

8	Cell Differentiation/ or Cellular Reprogramming/

9	(differentiat* or undifferentiat* or un-differentiat* or reprogram* or matur*).ab,kf,ti.

10	8 or 9

11	4 and 7 and 10

Search link: https://ovidsp.ovid.com/ovidweb.cgi?T=JS&NEWS=N&PAGE=main&SHAREDSEARCHID=1KtoDSlXecPEAMHh09FTXWyPMUKa0spbcbNm0lpvlyp4Bc4XuGeAlcNAANBQ3n2H1

////////////////////////////////////////////////////////

Embase (Elsevier)

Run Date: 06/12/23

Results: 355

Details:

Sources: Embase, MEDLINE, Preprints

Query: (mirna* OR microrna* OR 'micro rna*' OR mirnome* OR 'mir 2110' OR 'mir 340' OR 'mir 506 3p' OR 'mir 124 3p' OR 'mir 61' OR 'mir 10a' OR 'mir 10b' OR 'mir 14q32' OR 'mir 15a' OR 'mir 15b' OR 'mir 16' OR 'mir 17 92' OR mir137 OR 'mir 410' OR 'mir 487b' OR 'mir 490 5p' OR 'mir 558' OR 'mir 9 3p' OR 'mir 218 5p' OR 'mir 1538' OR 'microrna'/exp OR 'microrna') AND (neuroblastoma* OR ganglioneuroblastoma* OR 'neuroblastoma'/exp OR 'neuroblastoma') AND ('differentiation' OR 'undifferentiation' OR 'reprogramming' OR 'maturation' OR 'cell differentiation'/exp)
